# Long-term forecast for antibacterial drug consumption in Germany using ARIMA models

**DOI:** 10.1007/s00210-024-03721-4

**Published:** 2025-01-04

**Authors:** Lilly Josephine Bindel, Roland Seifert

**Affiliations:** Hannover Medical School, Institute of Pharmacology, D-30625 Hannover, Germany

**Keywords:** Antimicrobial consumption, Antibiotic, Antibacterial drug, AMR, Antibiotic prescription, Germany, Arzneiverordnungs report, Surveillance, Antibiotic stewardship, Rational prescribing behavior, Irrational prescribing behavior, Forecast, ARIMA

## Abstract

The increasing supply shortages of antibacterial drugs presents significant challenges to public health in Germany. This study aims to predict the future consumption of the ten most prescribed antibacterial drugs in Germany up to 2040 using ARIMA (Auto Regressive Integrated Moving Average) models, based on historical prescription data. This analysis also evaluates the plausibility of the forecasts. Our findings represent one of the first long-term national forecasts for antibacterial drug consumption. ARIMA(**0,1,0**), a random walk model with drift, is the best-fitting model to capture trends across all antibacterial drugs. While more complex models offer greater detail, they seem less suitable for long-term forecasting. In a short-term forecast of 5 and 10 years, predictions between significant models vary very little. Predictions indicate increasing DDD-prescriptions for amoxicillin, cefuroxime axetil, amoxicillin clavulanic acid, clindamycin, azithromycin, nitrofurantoin, and ciprofloxacin, while declines are forecasted for doxycycline, phenoxymethylpenicillin, and sulfamethoxazole-trimethoprim. The reliability of the predictions varies. Forecasts for azithromycin, phenoxymethylpenicillin, and sulfamethoxazole-trimethoprim are likely accurate, whereas uncertainties exist for doxycycline, amoxicillin clavulanic acid, nitrofurantoin, and ciprofloxacin, though general trends appear valid. Potential discrepancies may arise in the predictions for amoxicillin, cefuroxime axetil, and clindamycin. These forecasts highlight the urgent need for proactive healthcare planning to prevent future shortages, a problem underscored by recent supply disruptions in Germany. Future research should extend this analysis to the development of bacterial resistance and other frequently used drug classes.

**Supplementary Information:**

The online version contains supplementary material available at 10.1007/s00210-024-03721-4.

## Introduction

There are several alarming trends concerning antibacterial drugs that threaten both their efficacy and availability in treating bacterial infections. One major issue is the rapid global rise in bacterial resistance (UN 2024), which has already placed significant burdens on health systems worldwide, including Germany (IHME 2024). This trend is projected to escalate, potentially leading to millions of deaths globally in the coming years (Antimicrobial Resistance Collaborators 2024). Although global initiatives to combat antimicrobial resistance are beginning to be planned (UN 2024), addressing this challenge will require long-term, concerted efforts.

As the ability to effective treatment of bacterial infections diminishes, it is important to ensure that the most appropriate antibacterial drug (Bindel and Seifert 2024a) is available to treat the respective infection. However, this has not always been the case. Many frequently used antibacterial drugs have faced prolonged and recurring supply shortages in recent years (BfArM 2024; Tagesschau 2024; Berndt 2024), beside evidence of irrational prescribing behavior (Bindel and Seifert 2024b, c). Recently, all of the analyzed drugs have faced a delivery shortage (Table 1). These shortages contribute to higher risks of treatment failures, support irrational prescribing behavior and an acceleration of bacterial resistance, further complicating a successful treatment.

**Table 1:** Official announcements of supply shortages by the BfArM for Germany (PharmNet.Bund 2024). Information is given whether there is a current shortage (status: October 12, 2024). If the antibacterial drug is available recently, the last period of supply shortage is mentioned. Antibacterial drugs that are currently in short supply are shown in italic. Drugs that are currently available but have experienced shortages are shown in bold. Notably, all ten antibacterial drugs analyzed have experienced a supply shortage within the last 2 years

Ranking	Antibacterial drug	Current delivery shortage (actuality: October 12, 2024)	Period of last delivery shortage (year)	Source
1	*Amoxicillin*	Yes		PharmNet.Bund 2024
2	**Cefuroxime axetil**	No	Yes (2023)	PharmNet.Bund 2024; Apotheke Adhoc 2023
3	*Doxycycline*	Yes		Tagesschau 2024; Berndt 2024
4	**Amoxicillin clavulanic acid**	No	Yes (2023)	PharmNet.Bund 2024; BfArM 2023
5	*Clindamycin*	Yes		PharmNet.Bund 2024
6	*Azithromycin*	Yes		PharmNet.Bund 2024; Berndt 2024
7	*Phenoxymethylpenicillin*	Yes		PharmNet.Bund 2024
8	**Sulfamethoxazole-Trimethoprim**	No	Yes (2024)	PharmNet.Bund 2024; Apotheke Adhoc 2024
9	**Nitrofurantoin**	No	Yes (2022)	PharmNet.Bund 2024
10	*Ciprofloxacin*	Yes		PharmNet.Bund 2024

In response to these challenges, our study aims to predict the future consumption of the most commonly used antibacterial drugs in Germany through 2040, based on historical prescription data. We used the ARIMA model to forecast predictions and trends and evaluated its reliability. Explanations of parameters, models, and fit metrics can be found in Tables 2, 3 and 4. Earlier research of ourselves investigated the development and potential influence of external factors (Bindel and Seifert 2024a, b, c, d) . These findings were used to interpret predictions of the ARIMA model. To our knowledge, our findings are the first long-time nationwide prediction for a number of popular antibacterial drugs. Although ARIMA models are frequently used in scientific research (see Table 5), there is a lack of long-term predictions and its assessment.

**Table 2:** Explanation of parameters for an ARIMA(**p,d,q**) model (Hyndman and Athanasopoulos 2018; NIST/SEMATECH 2012)

Parameter	Description	Value range	Interpretation	Influence on the model	Process of determination
*p*	Autoregressive term—specifies the number of lagged observations included in the model	Non-negative integers (e.g., 0, 1, 2, …)	Determines how many past values contribute the current prediction	Higher values increase model reliance on past values, potentially increasing complexity	Autocorrelation, PACF plot, significant lags
*d*	Differencing order—gives the number of performed differentiations of data to achieve stationarity	Non-negative integers (e.g., 0, 1, 2, …)	Ensures a stationary time series by removing trend	Higher values may lead to over-differencing, potentially removing important data structure	Autocorrelation, examination of stationarity of data in ACF and PACF plot
*q*	Moving average term—refers to the number of lags, forecast errors included in the model	Non-negative integers (e.g., 0, 1, 2, …)	Specifies how many forecast errors influence current predictions	Larger values ensure the use of more past error terms, potentially increasing complexity	Autocorrelation, ACF plot, significant lags

**Table 3:** Overview about variations of parameters in ARIMA models (Nau 2020; Box et al. 2015; Hyndman and Athanasopoulos 2018)

ARIMA model	Description	Advantages	Disadvantages	Assessment
ARIMA(0,1,0)	Simple random walk model	Easy to interpret, suitable for non-stationary data	Does not account for seasonality or autocorrelation	Simple baseline model, useful for trends but lacks depth and ignores autocorrelation
ARIMA(1,0,0)	Autoregressive model (ar)	Captures trends, handles autocorrelation	Assumes stationarity, misses shocks	Best for stationary series with trends, struggles with non-stationary data or sudden changes
ARIMA(0,1,1)	Integrated moving average	Smooths short-term fluctuations, handles non-stationary data	Limited flexibility	Effective for moderate trends, but insufficient for more complex patterns
ARIMA(1,1,0)	Autoregressive integrated	Balances trend and autocorrelation, fits non-stationary data	Assumes linear relationships, may overfit	Good for capturing trends and autocorrelation but may miss shocks or rapid changes in data
ARIMA(0,1,2)	Moving average with two lags	Handles noise and short-term shocks	Complex, risk of overfitting	Handles random noise well, but its complexity can lead to overfitting
ARIMA(1,1,1)	Full arima model with one lag each	Captures both trend and random noise, versatile	Computationally intensive, risk of overfitting	Suitable for complex data with trends and noise but can overfit
Seasonal ARIMA (SARIMA)	Seasonal arima	Handles seasonality, trends, and autocorrelation	Complex to implement, requires large datasets, only useful if data is seasonal	Good choice for seasonal patterns but demands more data

**Table 4:** Explanation of fit metrics (Hyndman and Athanasopoulos 2018; NIST/SEMATECH 2012)

Fit metric	Description	Value range	Interpretation	Use case	Typical values for good fit	Limitations
Stationary *R*-squared	Measures how well the model fits for the stationary part of the time series	0–1 (higher is better)	High values indicate better fit, compares model on a stationary scale	Useful when dealing with non-stationary data that has been differenced	Close to 1 suggests a good fit for stationary data	May not be comparable across models with different degrees of differentiations
*R*-squared	Indicates proportion of the variance in the dependent variable that is predictable from independent variables	0–1 (higher is better)	High values indicate better fit, refers to the explanatory power of the model over the data	General model assessment, useful for comparing model strength	Close to 1 for models with high explanatory power	Could be misleading for time series due to potential autocorrelation in residuals
RMSE	Root mean square error, measuring the average magnitude of residuals	Non-negative real numbers	Low values indicate better fit, measures how close predicted values are to observed values	Assessment of predictive accuracy	Close to zero in dicates minimal prediction error	Sensitive to outliers, which can inflate the error
MAPE	Mean absolute percentage error, measuring accuracy as a percentage	0.0–100.0%	Low percentages indicate better accuracy, relative comparison across different scales	Helpful when relative error is more important than absolute error	Values below 10.0% are considered as good	Can mislead for small actual values, inflating the error
MaxAPE	Maximum absolute percentage error, shows the largest absolute percentage error	0.0–100.0%	Low values indicate better fit, shows the maximum difference between observed and predicted values in percentage	Useful for understanding the worst-case relative error	Lower values (close to zero) are better	Heavily influenced by single outliers, which may not represent overall fit
MAE	Mean absolute error, providing the average absolute difference between predicted and observed values	Non-negative real numbers	Low values indicate better fit, represents the average extent of errors	Useful for interpretation and comparison with RMSE	Close to zero indicates lower average error	Does not punish larger errors as much as RMSE, potentially downplaying significant deviations
MaxAE	Maximum absolute error, indicating the largest absolute error observed	Non-negative real numbers	Low values indicate better fit, provides the worst-case absolute deviation from the actual value	Helps identify the largest error in prediction, provides insight into worst-case scenarios	Lower values (close to zero) are better	Heavily influenced by outliers, which may not reflect overall fit quality
Normalized BIC	Bayesian Information Criterion, adjusted for model comparison on a standardized scale	Real numbers (lower is better)	Low values indicate better model fit relative to complexity, penalizes overly complex models more heavily than AIC	Effective for comparing models, especially with different numbers of parameters	The lowest number among compared models is preferred	Risk of over-penalizing complexity, possibly leading to underfitting

**Table 5:** *S*election of available literature for predictions based on the ARIMA model

Publication	Analyzed time period	Region	Topic	Used model	Assessment of fit
Asencio et al. 2017	2008–2012	Spain, primary health care area	Bacterial resistance of E. coli	ARIMA (model unknown)	Good fit, enables future prediction
Colson et al. 2019	2000–2015	France, Italy, Spain, UK	Forecast of bacterial resistance for six pathogen-drug pairs	ARIMA(0,1,0), ARIMA(0,0,0), ARIMA(0,2,0), ARIMA(1,2,0), ARIMA(1,1,0)	Statistical good fit, but differs from expert judgment; need to assess forecasts with real-world data
Gharbi et al. 2015	2005–2014	UK, London, renal unit	Forecasting bacterial resistance by consumption rates	ARIMA (model unknown)	Provides a key warning indicator, suitable for routine surveillance
Hur et al. 2024	April 2013–December 2019	USA, California, Vacaville, hospital	Prediction of bacterial resistance for E. coli	ARIMA (model unknown)	Good fit, better than neural network and random forest models
Jeffrey et al. 2020	2012–2016; 2015–2018	Europe; UK	Forecast of consumption and bacterial resistance	“naive”	Accurate for Europe; UK data shows trend-seasonal forecasts are more accurate on a finer scale
Jian et al. 2022	2000–2019	China	Forecast of chronic kidney disease prevalence and economic burden	ARIMA(1,1,1)	Good fit, reliable, forecasts extend from 2019 to 2025
Jimenez et al. 2020	January 2009–January 2018	Spain, university hospital of Getafe	Forecast of bacterial resistance/future outbreaks for MRSA	seasonal ARIMA (MARIMA, VARMA)	Accurate monthly predictions for 1 to 3 steps ahead
Kari et al. 2023	January 2017–February 2022	Finland, outpatient setting	Consumption of antibacterial drugs during the COVID-pandemic	ARIMA (model unknown)	Good fit
Kim et al. 2023	2010–2021	USA, pigs, farms of food production	Forecast of bacterial resistance for E. coli, S. suis, Salmonella spp, P. multocida, B. bronchiseptica	SARIMA, ARMA	One data point per quarter, good fit; SARIMA outperforms ARMA and ARIMA for seasonal data, good for trends
Somyanonthanakul et al. 2022	March 2020–August 2021	Thailand, university hospital	COVID-19 predicting incidence	ARIMA(2,2,2)	Good fit, potential for accurate prediction
Shoko and Njuho 2022	March 5, 2020–July 15, 2021	South Africa, Zambia, Namibia	COVID-19 predicting incidence	ARIMA(11,1,11), ARIMA(11,1,9), 515 observations (one per day)	Good fit, best-fitting model consistent with tested forecasts over 15 days
Xie et al. 2020	2010–2018	China	Predicting trends in antibacterial use in outpatients	ARIMA (model unknown)	Good fit, recommended for short-term prediction

To ensure a safe, effective, and rational treatment of bacterial infections, it is essential that the best treatment options remain available. Healthcare stakeholders must take proactive steps to maintain adequate supplies of these drugs. With a long-term forecast, future consumption trends can be estimated, enabling healthcare systems to take necessary precautions and the supply shortages that occurred frequently in recent years.

## Materials and methods

### Setting and data collection of DDD-prescriptions

The analysis focuses on outpatient prescriptions of antibacterial drugs in Germany. Data of defined daily dose (DDD-) prescriptions is based on the Arzneiverordnungsreport (AVR, Drug prescription report) for the years 1985 to 2022 (Schwabe and Paffrath 1985, 1986, 1987, 1988, 1991, 1992, 1993, 1994, 1995, 1996, 1998, 1999, 2000, 2001, 2002, 2003, 2004, 2006, 2007, 2008, 2010a, b, 2011a, b, 2012, 2013, 2014, 2015, 2016, 2017; Schwabe, Paffrath and Anlauf 1989, 1990; Schwabe 1997; Schwabe, Paffrath, Ludwig and Klauber, 2018, 2019; Schwabe and Ludwig 2020; Ludwig, Mühlbauer and Seifert, 2021, 2023, 2024). Since we focused on outpatient prescriptions, only the general chapter “Antibiotika und Chemotherapeutika” (antibiotics and chemotherapeutics) was considered. Therefore, specialized subchapters like urology, dermatology, and ophthalmology were excluded. We only included antibacterial drugs, resulting in the exclusion of groups like antimycotics, antiretroviral drugs, and antivirals.

For a more focused analysis, we selected the ten most prescribed antibacterial drugs (**TOP10**) in 2022. The resulting data is on a yearly basis and non-seasonal; 37 time points are included, with the quantities expressed in millions for the entire German outpatient sector.

### Data preprocessing

The dataset required no cleaning or transformation, as it was complete, and all variables were on the same scale. However, a precondition for ARIMA modelling is data stationarity (Hyndman et al. 2018). Autocorrelation function (**ACF**) and partial autocorrelation function (**PACF**) tests were applied to each antibacterial drug in the dataset, revealing significant trends and confirming non-stationarity (Table S1, S3, S5, S7, S9, S11, S13, S15, S17, S19). As a result, differencing was necessary to remove these trends and ensures stationarity, which is essential for accurate predictions. Differencing allows the ARIMA model to capture temporal dependencies without the distortion caused by persistent trends, enabling more reliable forecasting. After that, significant lags in ACF and PACF were identified (Table S2, S4, S6, S8, S10, S12, S14, S16, S18, S20) to identify fitting parameters for the ARIMA model for each antibacterial drug.

### Model selection

The ARIMA model was selected for its versatility in handling various components of a time series, including autoregressive (**AR**) behavior, differencing (**I**), and moving average (**MA**) effects (Tables 2 and 3). This model is particularly well-suited to data such as ours, where past observations influence future values. ARIMA models are widely used in forecasting drug consumption trends and the development of bacterial resistance (Hyndman and Athanasopoulos 2018), as depicted in Table 3, and have been shown to perform comparably to advanced modelling methods like deep learning (Kontopoulou et al. 2023).

The ARIMA model, which stands for “Auto Regressive Integrated Moving Average”, consists of three key components. The autoregressive (**AR**) part accounts for the influence of previous observations on the current value. The differencing (**I**) component helps in making the series stationary by removing trends or seasonality. Finally, the moving average (**MA**) component captures the dependency between an observation and past forecast errors (Nau 2020; Box et al. 2015).

The general form of an ARIMA(***p*,*d*,*q***) model is given by:

$$\widehat{y}t=\mu +\phi 1yt-1+\cdots +\phi pyt-p-\theta qet-q$$

In this equation, $\widehat{y}t$ represents the value of the series at time, $t, p$ refers to the number of autoregressive terms, *d* is the degree of differencing applied to make the series stationary, and *q* is the number of lagged forecast errors in the moving average model. The error term $et$ captures the random shocks that cannot be explained by the model (Nau 2020; Box et al. 2015).

For our analysis, the differencing parameter *d* = 1 was selected to remove the trend and achieve stationarity in the time series. The autoregressive term *p* and the moving average term *q* were determined by analyzing the autocorrelation function (**ACF**) and partial autocorrelation function (**PACF**) plots, which highlight significant lag structures in the data. In some models created, the AR or MA is < 1. In this case, the model includes all delays. For example, the variation for amoxicillin clavulanic acid with ARIMA(0,1,5), all five delays (MA1, MA2, MA3, MA4, MA5) are included.

An important variation of the ARIMA model is ARIMA(**0,1,0**), which was used in our analysis. This model applies first-order differencing to remove trends, but does not include autoregressive or moving average components. Essentially, ARIMA(**0,1,0**) is a random walk model with drift and comparable with a linear regression model, where future values depend solely on the current value and a constant trend term. This model is suitable for processes where the best prediction is simply the most recent observation plus a trend (Nau 2020).

### Model specification

For each antibacterial drug, the ARIMA model was specified based on the previously determined parameters *p*, *d*, and *q*. Since ARIMA combines both autoregressive and moving average components, it is very effective for time series forecasting (Hyndman and Athanasopoulos 2018). However, a key limitation of the model is its assumption of linear relationships between time points, which may not account for sudden structural changes or nonlinear patterns in the data. In the case of clindamycin, for example, a level shift in 2012 was observed due to the inclusion of dental prescriptions (Bindel and Seifert 2024d). This structural change was addressed using automatic outlier detection, ensuring that the forecasts were not distorted. For all other drugs, no outlier detection was required.

### Model fitting and forecasting

**Fig. 1:**
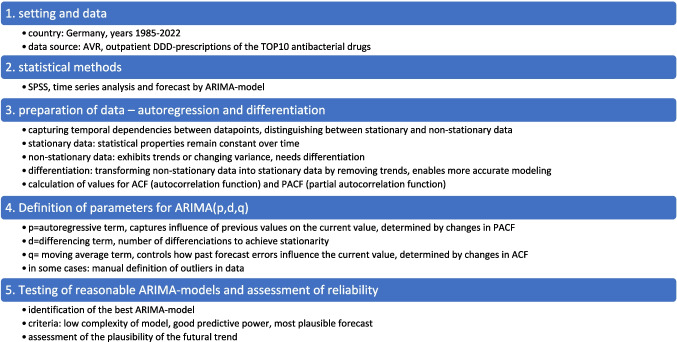
Methodical approach for time series analysis in SPSS with the ARIMA model to predict futural development of DDD-prescriptions for antibacterial drugs

After selecting and specifying the appropriate ARIMA model for each antibacterial drug, the models were created using SPSS. The goal was to minimize deviations between predicted and observed values, ensuring that the model captured the underlying trends as accurately as possible (NIST/SEMATECH 2012). Forecasts were generated up to the year 2040, providing long-term predictions for trends in DDD-prescriptions for each antibacterial drug. Furthermore, a 5- and 10-year prediction is displayed to provide a more accurate prediction in a shorter time period. Confidence intervals were also generated to highlight the uncertainty of these forecasts, offering a range in which future consumption values are expected to fall (Fig. 1).

### Assessment of fit metrics

To assess the performance of the ARIMA models, several fit metrics were used. This includes stationary *R*-squared, *R*-squared, RMSE (root mean squared error), MAPE (mean absolute percentage error), MAE (mean absolute error), and normalized BIC (Bayesian information criterion). A detailed explanation of each metric is provided in Table 4. These metrics offer a comprehensive evaluation of the models’ fit to the observed data and their predictive accuracy.

The most important results of our study are presented in Figs. 2, 3, 4, and 5 and Tables 1, 2, 3, 4, 5, 6, 7, 8 and 9. Additional information is available in the appendix, including supplemental Figures S1-S28 and Tables S1-S5. The methodological process is summarized in Fig. 1.

**Fig. 2:**
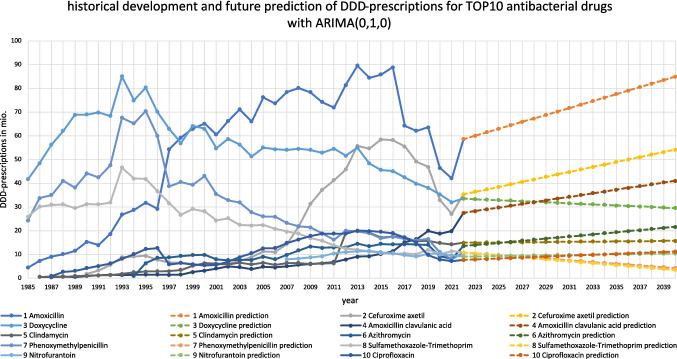
Overview on predictions of the analyzed TOP10 antibacterial drugs with the ARIMA(0,1,0) model

**Fig. 3:**
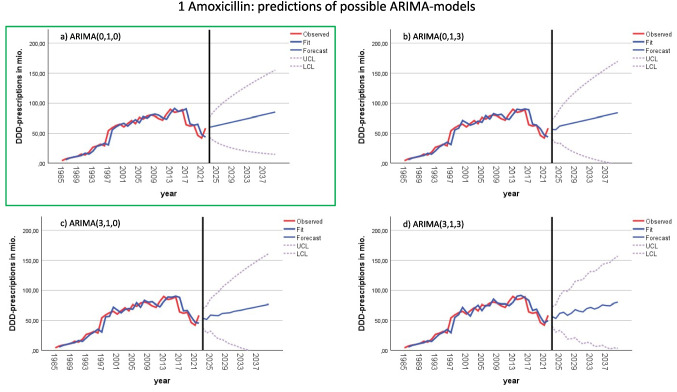
Predictions of future DDD-prescriptions of fitting ARIMA models for amoxicillin. Appropriate models include ARIMA (0,1,0) in **a**), ARIMA(0,1,3) in **b**), ARIMA(3,1,0) in **c**) and ARIMA (3,1,3) in **d**). The model with is considered as best-fitting is in a green box, being most reliable for forecasting future demand

**Fig. 4:**
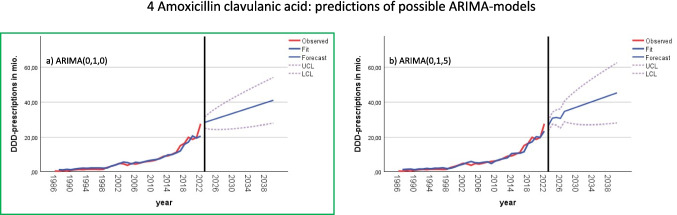
Predictions of future DDD-prescriptions of fitting ARIMA models for amoxicillin clavulanic acid. Appropriate models include ARIMA (0,1,0) in **a**) and ARIMA(0,1,5) in **b**). The model with is considered as best-fitting is in a green box, being most reliable for forecasting future demand

**Table 6:** Overview about fit values and prediction of the ARIMA(0,1,0) models for the ten analyzed antibacterial drugs. The table evaluates model fit based on several metrics. A good fit (italic) is indicated by high stationary *R*-squared (above 0.8), high *R*-squared (above 0.7), and low values for RMSE, MAPE, MaxAPE, MAE, MaxAE, and normalized BIC. A medium fit (bold) falls between 0.5 and 0.8 for stationary *R*-squared, 0.4–0.7 for* R*-squared, and moderate values (10–20%) for MAPE, MaxAPE, and MaxAE. A poor fit (bold italic) is characterized by low *R*-squared (below 0.4), high errors (MAPE, MaxAPE, and MaxAE above 20%), and the highest RMSE, MAE, and BIC values (Hyndman and Athanasopoulos 2018)

Antibacterial drug	1 Amoxicillin	2 Cefuroxime axetil	3 Doxycycline	4 Amoxicillin clavulanic acid	5 Clindamycin	6 Azithromycin	7 Phenoxymethylpenicillin	8 Sulfamethoxazole-Trimethoprim	9 Nitrofurantoin	10 Ciprofloxacin
Stationary *R*-squared	− ***2.22045E-16***	***1.11022E-16***	***0***	***2.22045E-16***	*0.89392461*	*** − 4.44089E-16***	***4.4409E-16***	0	− ***6.6613E-16***	4.4409E-16
*R*-squared	*0.903670863*	*0.943572847*	*0.804377936*	*0.948308601*	*0.9866927*	**0.488261064**	*0.8534672*	*0.89910489*	**0.56423801**	*0.87672532*
RMSE	***8.134240433***	4.715997782	5.495863856	1.523060828	0.7429444	1.950023742	6.20363765	3.28205376	0.64527587	1.96108356
MAPE	**10.86514237**	**16.51431905**	*7.040273763*	***31.17203993***	**10.1391198**	**13.70213771**	**12.5564422**	*7.50810563*	5.44134311	16.1602422
MaxAPE	***43.53192972***	***45.73518237***	**19.90779014**	***212.5***	***39.3650668***	***78.57142857***	***54.0959564***	***32.4208329***	**10.8333333**	122.315271
MAE	***5.265113221***	3.337304688	4.082395909	0.875	*0.53857144*	1.3	3.86880935	1.89393718	*0.53866667*	1.33257143
MaxAE	***26.06216216***	**15.046875**	**16.92162162**	*6.95*	*1.9457144*	*5.25*	***20.9351351***	**15.1081081**	*1.12666667*	*7.09428571*
Normalized BIC	***4.289757108***	3.210225272	3.505583942	0.940986218	− 0.39518374	1.454690399	3.74786405	2.47453112	− *0.69561801*	1.44857569
Outliers	No	No	No	No	Yes	No	No	No	No	No
Trend	Increasing	Increasing	Decreasing	Increasing	Slowly increasing	Increasing	Decreasing	Decreasing	Increasing	Increasing
Possibility for both decline and rise	Yes	Yes	Yes	No	Yes	Yes	Yes	Yes	Yes	Yes
Number of possible models	4	3	2	2	1	1	1	1	1	2
DDD in 2022	58.5	35.3	33.5	27.5	13.5	13.5	10.8	10.8	9.1	7.7
Predicted DDD for 2040	84.8	54.1	29.5	41	15.7	21.6	4.2	3.5	10.4	11.2
Relative change 2040 to 2022 in %	+ 45.0%	+ 52.4%	− 11.9%	+ 49.1%	+ 16.3%	+ 60.0%	− 61.1%	− 67.6%	+ 14.3%	+ 45.5%
Predicted UCL for 2040	154.8	95	76.8	54.1	22.1	38.6	57.6	31.7	16.3	28.1
Relative change 2040 to 2022 in %	+ 164.6%	+ 169.1%	+ 129.2%	+ 96.7%	+ 63.7%	+ 185.9%	+ 433.3%	+ 193.5%	+ 79.1%	+ 264.9%
Predicted LCL for 2040	14.8	13.3	0	27.9	9.3	4.6	0	0	4.5	0
Relative change 2040 to 2022 in %	− 74.7%	− 62.3%	− 100.0%	+ 0.7%	− 31.1%	− 65.9%	− 100.0%	− 100.0%	- 50.6%	- 100.0%
Range of LCL and UCL in %	239.3	231.4	229.2	97.4	94.8	251.8	533.3	293.5	129.7	364.9

## Results and discussion

### Choice of ARIMA model

Initially, all analyzed time series exhibited non-stationarity, indicating that trends influenced their properties, which could adversely affect the predictive accuracy. To address this, the data were transformed into a stationary time series through differentiation, thereby stabilizing the variance of the series (Hyndman and Athanasopoulos 2018). This transformation allowed the identification of significant lags in the differentiated ACF and PACF, which were then used to determine the parameters for the ARIMA models (Fig. S1-S20).

For several antibacterial drugs, a single ARIMA model emerged as the most appropriate. This was the case for clindamycin, azithromycin, phenoxymethylpenicillin, sulfamethoxazole-trimethoprim, and nitrofurantoin. None of them displayed any significant lags in the differentiated ACF and PACF. Consequently, after applying one differentiation, the time series for these drugs was best represented by the ARIMA(0,1,0) model. Curves and predictions for these antibacterial drugs can be found in Fig. 2 and S13-S17.

In some cases, certain lags were nearly significant but did not meet the threshold for statistical significance. To ensure that no relevant information was overlooked, alternative models were considered for these borderline cases. For example, amoxicillin exhibited a marginally significant lag at 3 in both ACF and PACF, leading to the exploration of ARIMA(0,1,0), ARIMA(0,1,3), ARIMA(3,1,0), and ARIMA(3,1,3) models (Fig. 3). Similarly, doxycycline showed a barely significant lag at 8 in the PACF, resulting in potential models ARIMA(0,1,0) and ARIMA(8,1,0), as depicted in Fig. S12. Amoxicillin clavulanic acid exhibited a narrowly significant lag at 5 in the ACF, leading to the consideration of ARIMA(0,1,0) and ARIMA(0,1,5) models (Fig. 4).

Significant lags, resulting in a variety of reliable ARIMA models, were identified for cefuroxime axetil and ciprofloxacin. Cefuroxime axetil demonstrated several significant lags, including lag 1 in PACF and lags 1, 8, and 11 in ACF. This results in possible models ARIMA(0,1,0), ARIMA(1,1,1), ARIMA(1,1,8), and ARIMA(1,1,11). Ciprofloxacin depicts a significant lag at 1 in both ACF and PACF, suggesting the models ARIMA(0,1,0) and ARIMA(1,1,1). Curves and predictions for both antibacterial drugs are depicted in Fig. 2, S11, and S12.

Outliers were not automatically detected, as the data are generally considered highly reliable, representing actual prescription figures collected by the WIdO (*wissenschaftliches Institut der AOK)*. Given that the data encompasses complete yearly records, it is assumed that all DDD-prescriptions were captured, with any fluctuations attributable to changes in prescribing behavior. However, an exception was made for clindamycin, where a shift in data collection in 2012, due to the inclusion of dental prescriptions, distorted the trend, resulting in an artificial increase. To mitigate the impact of this level shift on predictions, an outlier with a level shift was manually defined for clindamycin in 2012. Additionally, a sharp decrease in antibacterial prescriptions was observed around 2021 due to the COVID pandemic. Although this decrease had a strong impact on the curves, it was not defined as an outlier, as it reflected a real event. Nonetheless, it is possible that this event influenced the results, given its extraordinary measures (Fig. 2).

For all antibacterial drugs, the ARIMA (0,1,0) model was selected as the best-fitting model to predict future DDD-prescriptions. This model provides a reasonable outlook, with maximum possible values remaining within realistic bounds. Predictions and fit metrics for all antibacterial drugs for the model ARIMA(0,1,0) can be found in Table 6 and Fig. 2. In cases where multiple models were considered, ARIMA(0,1,0) was favored due to its superior Bayesian information criterion (BIC), as detailed in the supplementary materials. Utilizing the same model across all antibacterial drugs ensures the comparability of forecasts. The ARIMA(0,1,0) model, commonly known as a random walk model (Table 3), assumes that the time series follows “the last period’s value plus a constant representing the average change between periods” (Nau 2020). This approach is well-suited for modelling the development of DDD-prescriptions.

While more complex models might better capture underlying regularities and offer more precise forecasts—indicated by higher stationary *R*-squared and *R*-squared values—there is a significant risk of overfitting, where random fluctuations are mistakenly identified as patterns. Given the relatively limited dataset (37 data points), the model’s capacity is restricted. Therefore, greater emphasis should be placed on the reliability of trends rather than the precise prediction of specific DDD values in long-term periods, as indicated by the low value for the normalized BIC. For a shorter period of time, forecasts are considered to be more accurate than in long terms. Therefore, an overview of future consumption in a 5- and 10-year forecast is provided in Table S6. However, when multiple models are available, they provide insights into different potential outcomes, enabling a more nuanced evaluation of future forecasts.

### Antibacterial drugs with a predicted increasing trend: amoxicillin, cefuroxime axetil, amoxicillin clavulanic acid, clindamycin, azithromycin, nitrofurantoin, and ciprofloxacin

Seven of the ten antibacterial drugs analyzed—amoxicillin, cefuroxime axetil, amoxicillin-clavulanic acid, clindamycin, azithromycin, nitrofurantoin, and ciprofloxacin—are predicted to experience an increase in DDD-prescriptions in the future (Figs. 3, 4, S11, S13, S17, and S18). However, it is important to note that almost all these drugs could also see a decrease within their lower control limit (LCL), as depicted in Table 6. The exception is amoxicillin-clavulanic acid, for which the models rule out any potential for a future decline (Table S4).

Amoxicillin is expected to show a steady increase in DDD-prescriptions. The best-fitting model ARIMA(0,1,0) projects the largest rise, reaching 84.8 million DDD-prescriptions by 2040. Other models estimate slightly lower numbers, ranging from 76.6 to 83.8 million. Despite this upward trend, a decrease is still possible, with the ARIMA(0,1,0) model suggesting an LCL of 14.8 million in 2040. Some models, such as ARIMA(0,1,3) and ARIMA(3,1,0), even indicate the potential for prescriptions to drop to zero by 2040. On the other hand, a much higher consumption is also possible, with the ARIMA(0,1,0) model predicting an upper control limit (UCL) of 154.8 million DDD-prescriptions, and the ARIMA(0,1,3) model suggesting a peak at 169.3 million. The relative increase in prescriptions is projected to be between + 45.0 and + 30.9% by 2040, with extreme scenarios ranging from + 189.4 to − 100.0%. Specifically, for the ARIMA(0,1,0) model, the predicted range is + 164.6 to − 74.7% from 2022 to 2040.

Cefuroxime axetil is expected to experience a strong rebound, following a strong decline during the COVID pandemic. The ARIMA(0,1,0) model, which provides the most moderate prediction, estimates 54.1 million DDD-prescriptions by 2040. Other models suggest even higher numbers, with the ARIMA(1,1,8) model predicting up to 75.2 million DDD-prescriptions by 2040. Only the ARIMA(0,1,0) and ARIMA(1,1,1) models predict a possible decrease, with the latter even projecting zero DDD-prescriptions by 2035. However, other models forecast no decrease, with the lowest figures ranging from 29.2 million to 42.0 million. Regarding the UCL, the ARIMA(0,1,0) model provides a moderate estimate of 95.0 million DDD-prescriptions by 2040, while other models extend this to 133 million. The relative increase predicted by the ARIMA(0,1,0) model is + 54.1%, with other models suggesting a range from + 79.4 to 111.8%. The maximum possible decrease ranges from − 62.3 to − 100.0%, depending on the model.

Amoxicillin clavulanic acid is forecasted to continue its upward trend, with the ARIMA(0,1,0) model predicting 41.0 million DDD-prescriptions in 2040, and the ARIMA(0,1,5) model slightly higher at 45.3 million. Notably, both models exclude the possibility of a decrease, with an LCL of 27.9 million. The UCL ranges from 45.1 to 45.3 million, depending on the model. The predicted relative increase is + 49.1 to + 64.7%, with the highest projections between + 96.7 and + 127.6%, and the lowest between + 0.7 and + 1.8%.

Clindamycin is expected to see a modest increase, reaching 15.7 million DDD-prescriptions by 2040, according to the ARIMA(0,1,0) model. However, a larger increase up to 22.1 million (UCL) or a decrease to 9.3 million (LCL) is also possible, with a predicted relative change of + 16.3%, ranging from + 63.7 to − 31.1%.

For azithromycin, an increase is projected with the ARIMA(0,1,0) model estimating 21.6 million DDD-prescriptions by 2040. Like clindamycin, azithromycin’s trends could vary, with the UCL showing a continuation of the COVID spike, possibly leading to a plateau at 4.6 million DDD-prescriptions by 2040. Alternatively, a strong post-COVID increase could result in 38.6 million DDD-prescriptions. The predicted relative increase is + 60.0%, with potential extremes from + 185.9 to − 65.9% by 2040.

Nitrofurantoin is predicted to follow a moderate increase, reaching 10.4 million DDD-prescriptions by 2040, according to the ARIMA(0,1,0) model. However, this could range from 4.5 million (LCL) to 16.3 million (UCL), with a relative increase of + 14.3%, and possibilities ranging from + 79.1 to –50.6%.

Ciprofloxacin is expected to see a moderate increase, with the ARIMA(0,1,0) model predicting 11.2 million DDD-prescriptions in 2040. The ARIMA(1,1,1) model suggests a slightly higher figure of 11.7 million. Both models allow for potential decreases to zero DDD-prescriptions as early as 2025 or 2026, or increases up to 28.1 million (ARIMA(0,1,0)) or 39.4 million (ARIMA(1,1,1)) by 2040. The predicted relative increase is + 45.5 to + 51.9%, with possible extremes from + 264.9 to + 411.7%, and the lowest prediction at − 100.0%.

The strongest relative increase is depicted for azithromycin at + 60.0%, while amoxicillin is expected to see the largest absolute increase at + 26.3 million DDD-prescriptions. Conversely, nitrofurantoin is projected to have the lowest relative and absolute increase at + 14.3%. Ciprofloxacin shows the potential for the strongest relative increase (+ 264.9%) and the possibility of a complete reduction to zero. While a decrease to zero is possible for ciprofloxacin, only increasing trends are predicted for amoxicillin clavulanic acid. Cefuroxime axetil, although predicted by the ARIMA(0,1,0) model to possibly decrease, shows no potential for a decrease in two alternative models. Amoxicillin stands out for its potential for both the strongest absolute increase and decrease, likely due to its status as the most prescribed antibacterial drug in 2022. Azithromycin’s potential for the largest relative increase may be attributable to its smaller base volume in 2022, allowing for significant relative changes despite a more moderate absolute increase. Ciprofloxacin, with its wide range of possible outcomes, reflects a high level of uncertainty in its future trends.

### Antibacterial drugs with a predicted decreasing trend: doxycycline, phenoxymethylpenicillin, and sulfamethoxazole-trimethoprim

Three of the ten antibacterial drugs analyzed—doxycycline, phenoxymethylpenicillin, and sulfamethoxazole-trimethoprim—are predicted to experience a decrease in DDD-prescriptions in the future (Figs. S12, S15, and S16). Notably, all these drugs could see significant decreases within their lower control limit (LCL) (Table 6).

Doxycycline is forecasted to continue its downward trend, with the ARIMA(0,1,0) model predicting 29.5 million DDD-prescriptions by 2040, while the ARIMA(8,1,0) model predicts a slightly lower figure of 29.1 million (Table S3). Both models, however, indicate potential increases, with the ARIMA(0,1,0) model suggesting up to 76.8 million DDD-prescriptions and the ARIMA(8,1,0) model up to 85.7 million. Alternatively, a complete decrease to zero till 2040 is also possible. The predicted relative decrease for doxycycline ranges from − 11.9 to − 13.1%, with possible extreme increases (UCL) from + 129.2 to + 155.8%, while the lowest prediction suggests a total decline (− 100.0%).

Phenoxymethylpenicillin is expected to undergo a moderate decrease, with the ARIMA(0,1,0) model forecasting a reduction to 10.8 million DDD-prescriptions by 2040. However, predictions range widely, with a potential decrease to zero (LCL) or an increase up to 57.6 million (UCL). The relative decrease is predicted at − 61.1%, but possibilities range from a significant increase of + 433.3% to a complete decrease of –100.0%.

Sulfamethoxazole-trimethoprim is also expected to continue its downward trend, with the ARIMA(0,1,0) model predicting a reduction to 3.5 million DDD-prescriptions by 2040. The model also allows for a possible complete decline (LCL) to − 100.0%, or a substantial increase up to 31.7 million (UCL). The predicted relative decrease is − 67.6%, with a wide range of possible outcomes, from an increase of + 193.5% to a total decrease of − 100.0%.

Sulfamethoxazole-trimethoprim is projected to experience the strongest relative and absolute decrease, with a decline of − 67.6% and a reduction of 7.3 million DDD-prescriptions from 2022 to 2040. Doxycycline, on the other hand, shows the smallest relative and absolute decrease, with a reduction of − 11.9% and 4 million DDD-prescriptions over the same period. For all three drugs, a much more significant decrease, potentially reaching zero DDD-prescriptions by 2040, is possible. Conversely, an increase is also possible, particularly notable for phenoxymethylpenicillin, which could see an increase of up to + 433.3%. This indicates that the predictions for sulfamethoxazole-trimethoprim carry considerable uncertainty, allowing for a wide range of potential outcomes. In contrast, the prediction for doxycycline is more constrained, suggesting a moderate decrease with fewer possible variations.

### Reliability and evaluation of forecasts

Accurately predicting future DDD-prescriptions of antibacterial drugs requires both forecasting models and an interpretation of potential trends. Each antibacterial drug provides a range of possible future developments, allowing for interpretation and consideration of various scenarios. To assess the reliability of these forecasts, we evaluate model fit metrics and assess the plausibility of the predicted trends by analyzing historical developments and possible external influences.

The forecasts are evaluated using key fit metrics, including stationary *R*-squared, *R*-squared, RMSE, MAPE, MaxAPE, MAE, MaxAE, and normalized BIC (Table 6). Stationary *R*-squared measures the model’s ability to explain stationary components in the data, while *R*-squared reflects the overall explanatory power. Normalized BIC provides a balance between model fit and complexity, with lower values indicating a better fit that avoids overfitting.

Among the antibacterial drugs analyzed, clindamycin exhibits the highest *R*-squared (0.987) and stationary *R*-squared (0.894), indicating a strong model fit. In contrast, azithromycin shows the lowest *R*-squared (− 0.488), suggesting significant challenges in accurately modelling its future prescriptions. The best BIC value is observed for nitrofurantoin (− 0.696), indicating a well-fitting model with minimal overfitting risk, while amoxicillin has the worst BIC value (4.290), reflecting a less reliable forecast.

The variability in the fit metrics suggest that certain drugs can be predicted with greater accuracy than others. As seen in Table 6, clindamycin and nitrofurantoin display high *R*-squared values and low BIC scores, suggesting that the model reliably forecasts future prescriptions for these drugs. On the other hand, amoxicillin and azithromycin, with lower *R*-squared values and higher BIC scores, imply less reliable forecasts. These differences highlight the challenges of modelling certain drugs due to fluctuating prescription patterns or external factors that influence consumption, such as DDD-costs, bacterial resistance development, guideline recommendations, or prescribing behavior (Bindel and Seifert 2024a, b, c, d).

The relative changes predicted in prescription levels by 2040 provide additional insight into expected trends. Azithromycin is forecasted to show the largest relative increase (+ 60.0%), while sulfamethoxazole-trimethoprim is expected to experience the largest decrease (− 67.6%). Doxycycline shows the smallest predicted change (− 11.9%), indicating a relatively stable use.

Uncertainties in the forecasts is further highlighted by the upper and lower control limits (UCL and LCL) for each drug. Phenoxymethylpenicillin shows the widest range of potential outcomes, with an upper limit of + 433.3% and the possibility of a − 100% decline. In contrast, clindamycin has the narrowest range, suggesting greater confidence in its forecasted increase (+ 63.7%).

When comparing the reliability of short-term versus long-term forecasts for antibacterial drugs with significant lags, no noteworthy differences emerge in the selection of the best-fitting ARIMA model. Doxycycline and ciprofloxacin were chosen as case studies, as these drugs exhibit significant lags in ACF and PACF; therefore, it is most likely that here another model might fit better for another set of prediction range. However, an examination of the 5-year and 10-year-predictions reveals only minor variations in forecasted consumption (5.0% and 1.3% for doxycycline, and 0.0% and 3.1% for ciprofloxacin), as depicted in Table 7. Notably, the more complex models display a broader range between the UCL and LCL for both drugs. Therefore, it is assumed that the ARIMA(0,1,0) model is the best fit for both short-term and long-term predictions. Nevertheless, while the prediction of a shorter period of time (Table S6) can be considered as reliable within an antibacterial drug considered to have an accurate model, there are some concerns about the ability to make accurate long-term projections to 2040. Therefore, long-term projections should be understood as focusing on the trend rather than the actual values.

**Table 7:** Evaluation of short-term predictions for doxycycline and ciprofloxacin. The 5-year and 10-year forecasts of DDD-prescriptions, UCL, and LCL are shown. In addition to an assessment of the absolute and relative changes in consumption between 2022 and the forecast, the two available models are compared for each antibacterial drug by showing the absolute and relative difference in their prediction

Antibacterial drug	3 Doxycycline		10 Ciprofloxacin	
Model	ARIMA(0,1,0)	ARIMA(8,1,0)	ARIMA(0,1,0)	ARIMA(1,1,1)
DDD-prescriptions in million in 2022	33.5		7.7	
Predicted DDD-prescriptions in million for 2027	32.4	30.8	8.7	8.7
Relative change 2027 to 2022 in %	− 3.3%	− 8.1%	+ 13.0%	+ 12.9%
Predicted UCL for 2027	57.3	54.5	17.6	21.2
Relative change 2027 to 2022 in %	+ 71.0%	+ 62.5%	+ 128.6%	+ 175.3%
Predicted LCL for 2027	7.5	4.2	0	0
Relative change 2027 to 2022 in %	− 77.6%	− 87.4%	− 100.0%	− 100.0%
Absolute difference between predictions	1.6		0	
Relative difference between predictions	5.0%		0.0%	
Predicted DDD-prescriptions in million for 2032	31.3	31.7	9.6	9.9
Relative change 2032 to 2022 in %	− 6.6%	− 5.4%	+ 24.7%	+ 28.6%
Predicted UCL for 2032	66.5	75.1	22.2	29.5
Relative change 2032 to 2022 in %	+ 98.5%	+ 124.0%	+ 188.3%	+ 283.1%
Predicted LCL for 2032	0	0	0	0
Relative change 2032 to 2022 in %	− 100.0%	− 100.0%	− 100.0%	− 100.0%
Absolute difference between predictions of possible models	0.4		0.3	
Relative difference between predictions of possible models	1.3%		3.1%	

Several external factors may contribute to the predicted increases or decreases in drug prescriptions. The increase in DDD-prescriptions for azithromycin could be attributed to its broad-spectrum effectiveness and potential for new therapeutic indications (Abdulrazak et al. 2017; Welte 2019). Conversely, the expected decline in sulfamethoxazole-trimethoprim may be a result of growing bacterial resistance (Bindel and Seifert 2024a, d) and concerns regarding side effects like allergic reactions (Serrano-Arias et al. 2023), leading to updated guidelines recommending against its use in some cases (DAZ 2012). The moderate increase predicted for nitrofurantoin (+ 14.3%) may be due to its continued effectiveness in treating urinary tract infections and its recommendation as a first-line therapy in guidelines (AWMF 2024; Bindel and Seifert 2024d), despite the availability of alternative treatments (Dos Santos et al. 2021).

**Fig. 5:**
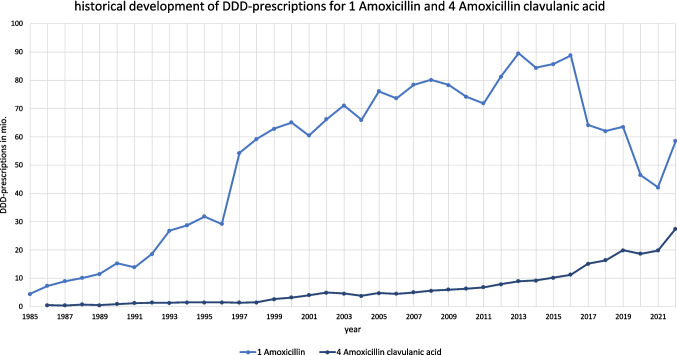
Development of DDD-prescriptions for amoxicillin and amoxicillin from 1985 to 2022. Their consumption is depicted separately, as their development is linked to each other (Bindel and Seifert 2024d)

In some cases, discrepancies between the model predictions and real-world expectations exist. For amoxicillin, the ARIMA(0,1,0) model suggests a potential increase in future consumption (+ 45.0%), although there are reasons that this forecast should be interpreted with caution. As one of the most frequently prescribed antibacterial drugs, amoxicillin has experienced fluctuating trends (see Figs. 2, 3, and 5), particularly due to competition with amoxicillin clavulanic acid (Bindel and Seifert 2024a), which is predicted to increase moderately (+ 49.1%). In Fig. 5, the development of DDD-prescriptions for amoxicillin and amoxicillin clavulanic acid is separately illustrated. This competition may make the future of amoxicillin prescriptions uncertain. While the model forecasts an increase, the actual trend could shift towards a decrease if prescribing preferences continue to favor amoxicillin clavulanic acid. Cefuroxime axetil, another drug projected to increase (+ 52.4%), experienced a post-pandemic increase. However, its declining trend in recent years, coupled with changes in its indications and removals of its recommendation in guidelines (AWMF 2024), raises doubts about the predicted increase. It is plausible that the model overestimates this rebound, and a more conservative outlook might suggest a continued decline.

Clindamycin presents a unique case, as the model forecasts a slow increase despite its long-term downward trend, exacerbated by the COVID pandemic. While the forecast suggests a potential rebound, this is likely an overestimation, given persistent factors such as side effects (Miller et al. 2023) and better treatment options due to a comparably high bacterial resistance (Tolksdorf et al. 2022), both of which are driving its decline.

Overall, the ARIMA(0,1,0) model provides reliable predictions for certain drugs, particularly those with consistent prescription patterns such as amoxicillin clavulanic acid, clindamycin, sulfamethoxazole-trimethoprim, and nitrofurantoin. However, for drugs like amoxicillin and cefuroxime axetil, where trends are more volatile or subject to external influences, e.g., changes in prescription behavior as happened in the COVID pandemic or strong impacts of cost changes (Bindel and Seifert 2024a, b, c), the forecasts should be interpreted with caution. Further analysis may be necessary as additional data becomes available. Therefore, a more precise analysis could be performed if the predictions are actualized with the data of current years.

In conclusion, the predictions for azithromycin, phenoxymethylpenicillin, and sulfamethoxazole-trimethoprim are likely accurate. However, some uncertainties exist regarding the future DDD-prescriptions of doxycycline, amoxicillin clavulanic acid, nitrofurantoin and ciprofloxacin, though the general trends seem correct. For amoxicillin and cefuroxime axetil, there may be discrepancies between the predictions and what is plausible, while the forecasted trend for clindamycin is likely overestimated.

### Limitations

The analysis presented in this study is based on data from the Arzneiverordnungsreport. As only outpatient prescriptions of the GKV system are included, no assessment can be made regarding prescriptions in hospitals or via private health insurance. As the data are based on developments in Germany, they are not directly transferable to other countries. No differentiation was made according to age or region.

Data collection for DDD-prescriptions depended on the design of the chapter under consideration. Changes in the structure of the sections over the years led to a bias for clindamycin in 2012 (Schwabe and Paffrath 2013). An automatic detection of outliers was not performed, since data was considered reliable and without errors. Nevertheless, random fluctuations could potentially influence the accuracy of the predictions.

Generalizations are restricted by the limited number of examined antibacterial drugs, pathogens, and factors considered. Data of DDD-prescriptions include a relatively small number of data points when aiming to use an ARIMA model. There may be other factors influencing the outcomes studied that were not included in the modelling process, their extent of influence remaining uncertain. Predictions can only be made based on current knowledge; therefore, unforeseen future developments cannot be anticipated. The further into the future the prediction is made, the wider the confidence interval and therefore the potential outcome, and the more the uncertainties increase.

Certain criteria were set for the statistical methods used (see Table 6). A simple model was chosen as the best-fitting approach one to prevent the risk of overfitting. Changing these parameters or priorities may lead to different conclusions.

## Conclusions

The prediction of future DDD-prescriptions was successful for all analyzed antibacterial drugs, though the accuracy varied (see Table 6 and Fig. 2). Reliable predictions were achieved for azithromycin (increasing), phenoxymethylpenicillin (declining), and sulfamethoxazole-trimethoprim (declining), while the predictions for doxycycline (declining), amoxicillin clavulanic acid (increasing), nitrofurantoin (increasing), and ciprofloxacin (increasing) captured the general trends but were less precise in their specific values. Some drugs, such as amoxicillin (increasing) and cefuroxime axetil (increasing), may show discrepancies between predicted and real-world values, being even more pronounced for clindamycin (increasing).

For an accurate prediction, the data structure is very important. It appeared that continuing curves are easier to model than developments with many spikes or abrupt changes, leading to instability in ARIMA models. The presence of outliers, as happened in clindamycin, can distort predictions, although these fluctuations often reflect real-world events, such as the COVID pandemic, and cannot simply be excluded. Moreover, the number of available data points is important. Despite spanning a long period from 1985 to 2022, the dataset includes only 37 data points, which is relatively low for robust mathematical modelling. This limited dataset impacts the model’s accuracy and helps explain discrepancies in the fit metrics. Conclusively, long-term predictions can be used to estimate a trend rather than predicting accurate consumption volumes, while in a short-term, the forecast can be seen as reliable in antibacterial drugs with a model considered as accurate.

The reliability of the predictions differs between antibacterial drugs, as reflected in the fit metrics. External factors, such as unexpected developments in bacterial resistance or sudden shifts in prescribing behavior due to changes in cost or medical guidelines, introduce uncertainty. The ARIMA model’s reliability is predicated on the assumption that conditions remain stable, making it less effective when forecasting within unpredictable or volatile conditions. The emerging uncertainty can be seen in the wide confidence intervals, enabling several potential outcomes.

The choice of the best-fitting ARIMA model has to balance between a detailed short-time prediction against a reliable long-term trend. Since our focus is on long-term developments, we selected the simple ARIMA(0,1,0) model, which performed well across all antibacterial drugs. This enabled a generalization and a comparability of forecasts between the analyzed drugs. Although more complex models offer more precise short-term predictions, they tend to be comparably accurate, but less plausible for long-term predictions. The simple model offers a moderate prediction with plausible ranges. In more complex models, there is a risk of overfitting, with fluctuations being interpreted as significant rules. In published literature, simple models are often used too (Jian et al. 2022; Colson et al. 2019).

ARIMA models offer both advantages and disadvantages (see Table 8). One major benefit is their ability to handle non-stationary data through differencing, an important feature given that initial tests showed non-stationarity. Another advantage is the transparency of ARIMA models. Their linear structure makes it easy to interpret how past values and errors influence future predictions, which is especially valuable in forecasting drug usage trends.

**Table 8:** Comparison of advantages and limitations of the ARIMA model, making it possible to evaluate whether the model is appropriate for time series forecasting of antibacterial drug consumption (Kontopoulou et al. 2023; Box et al. 2015; Hyndman and Athanasopoulos 2018)

Category	Advantages	Disadvantages
Identification of patterns and trends	Highly effective in modelling and forecasting time series with linear patterns or trends	Assumes a linear relationship, which may not be appropriate for nonlinear patterns or sudden structural breaks in the data
Stationarity adjustment	Can handle non-stationary data by applying differencing to achieve stationarity	Differencing can sometimes lead to over-differencing, removing important data structure
Integration of past values	Includes both past values (autoregressive part) and past errors (moving average part) for accurate forecasting	Does not account for external variables that might have an influence
Software availability	Widely used and supported by most statistical software	Choice of the correct parameters (*p*, *d*,* q*) requires careful analysis
Model complexity	Provides a balance between model complexity and fit, avoiding overfitting with an appropriate selection of parameters	High parameter complexity of *p*,* d*, or *q* values can lead to overfitting and unnecessary model complexity
Length of predictability	Good performance in short-term forecasting due to its reliance on recent data and trends	Long-term forecasts may be less accurate, as ARIMA relies heavily on past trends, which may change over time
Model transparency	Easy interpretation in terms of how past values and errors influence future predictions	Interpretation becomes more difficult in models with a high complexity
Handling of seasonal data	Simple models work well with deseasonalized data	Struggles with highly seasonal data, leading to the necessary of modifications like SARIMA for seasonal patterns
Performance with a small number of data points	Ability to work with small datasets as long as there is enough information for parameter estimation	In small datasets with complex structures, ARIMA may not capture enough data patterns, leading to reduced accuracy

However, a significant limitation of ARIMA is its assumption of linear relationships between time points, which may not fully capture the complexities of drug prescription patterns. Moreover, the chosen ARIMA(0,1,0) model, while comparable to linear regression models, shares some characteristics of deterministic models due to its simplicity. However, it remains fundamentally a stochastic prediction, as the random error term (*et*) directly influences future values and introduces uncertainty, which is reflected in the confidence intervals. These intervals are essential for assessing the plausibility of forecasts. While the structure of ARIMA(0,1,0) is linear, its reliance on the random component distinguishes it from a purely deterministic model, which would allow for exact predictions without uncertainty. The choice of ARIMA(0,1,0) was based on its plausibility and performance compared to more complex ARIMA models. After testing various alternatives, the simpler model proved to be the most realistic and suitable for the data. Since it was initially unclear whether the time series could be adequately represented using a purely linear approach, we adopted a comprehensive methodology instead of relying solely on linear regression models.

While ARIMA performs well for short-term predictions, its accuracy diminishes in long-term forecasts due to its reliance on historical trends (Hyndman and Athanasopoulos 2018). In Table 9, an overview and assessment of literature regarding the prediction of future consumption are provided.

**Table 9:** Selection of available literature for the prediction of consumptions, based on ARIMA models. Beside general information, the reliability and fit of the respective forecast is assessed

Publication	Analyzed time period	Region	Topic	Used model	Assessment of fit
Kari et al. 2023	January 2017–February 2022	Finland, outpatient setting	Consumption of antibacterial drugs during the COVID-19 pandemic	Unknown	Reported fit is good, but lack of model details limits evaluation
Jeffrey et al. 2020	2012–2016; 2015–2018	Europe; UK	Forecast of consumption and bacterial resistance	“naive”	Accurate for Europe, but less accurate for UK; limited robustness
Xie et al. 2020	2010–2018	China	Predicting trends in antibacterial use in outpatients	ARIMA(0,1,1)	Good for short-term predictions; appropriate for non-stationary data
Jian et al. 2022	2000–2019	China	Forecast of chronic kidney disease prevalence and economic burden	ARIMA(1,1,1)	Reliable
Linnér et al. 2020	2007–2018	Stockholm	Forecasting pharmaceutical expenditure in healthcare	ARIMA(1,0,1)	Good fit, but struggles with sudden changes
Rathipriya et al. 2022	2014–2019	Multiple regions	Forecasting drug demand	ARIMA, LSTM, shallow NN	ARIMA shows moderate fit, but machine learning models outperform it in complex datasets

The range of predictions are more important than exact predictions for purpose of a long-time span forecast. Literature research opts for the opinion that accurate predictions can only be ensured for a short time and predictions have to be updated (Shoko and Njuho 2022; Xie et al. 2020; Somyanonthanakul et al. 2022; Jimenez et al. 2020). Having a look at different models, variations in developments can be seen, which enables to be aware of different options in consumption and trends. Summarized, the ARIMA model is reliable for our dataset. Literature research confirms the usefulness of the chosen mathematical procedure for predicting future drug consumption (Asencio et al. 2017; Hur et al. 2024; Jian et al. 2022; Kim et al. 2023; Linnér et al. 2020). It also seems appropriate for the behavior of drug consumption (Kari et al. 2023; Jeffrey et al. 2020; Rathipriya et al. 2023). The fit metrics used in this study demonstrate that ARIMA models offer reliable predictions for short-term DDD-prescription trends. However, certain limitations—particularly the inability to capture seasonal patterns and the sensitivity to outliers—were evident in our case and already reported in earlier literature research (Jeffrey et al. 2020; Linnér et al. 2020). It is possible that expert opinions can differ from mathematical predictions (Colson et al. 2019). This strengthens the need for a detailed analysis of external factors and an assessment of reliability.

Given the goal to forecast future drug demand, it is essential for actors of the health system ensure sufficient capacity to maintain availability. Therefore, all stake holders of the health system have to get a knowledge about the future needs to plan for a long time and handle out contracts with the pharmaceutical industry. The failure to do so has led to persistent shortages in nearly all of the analyzed antibacterial drugs in recent years (BfArM 2024; Tagesschau 2024; Berndt 2024) (Table 1). Information on current shortages is accessible though the database provided by BfArM (https://anwendungen.pharmnet-bund.de/lieferengpassmeldungen/faces/public/meldungen.xhtml). These shortages not only pose a serious risk to patient care but also contribute to irrational prescribing behaviors and growing bacterial resistance, as non-optimal alternatives are used when preferred treatments are unavailable. The issue is likely to worsen in the future, underscoring the need for long-term planning. Our ARIMA-based predictions can serve as an early warning system (Gharbi et al. 2015), enabling proactive measures to enhance drug availability and prevent further shortages. Especially for the predicted rising consumptions in amoxicillin, cefuroxime axetil, amoxicillin clavulanic acid, azithromycin, nitrofurantoin, and ciprofloxacin, future availability for even higher DDD-prescriptions has to be ensured. These forecasts are essential for maintaining effective and safe treatments.

### Further perspectives

Further research is needed to confirm our findings and to expand the analysis to include more antibacterial drugs and parameters. As new data becomes available, there should happen a check of preciseness with optional adjusting. Besides a forecast of future consumption, a prediction for bacterial resistance development is urgently needed, particularly for problematic pathogens. Furthermore, an analysis of the total consumption of the antibacterial drug class for different countries is important in order to assess the international trends.

Extending the study to further drug classes which are likely to follow an increasing trend is important to ensure their availability. This is especially important for the most frequently used drugs in the German healthcare system. Therefore, a long-time prediction for the most prescribed drugs in Germany is urgently needed.

## Supplementary Information

Below is the link to the electronic supplementary material.

Supplementary file1 (DOCX 8.30 MB)

## Data Availability

All source data for this study are available upon reasonable request from the authors.

## Abbreviations

ACF: Autocorrelation function
ARIMA: Autoregressive integrated moving average model (mathematical model for time series analysis)
AVR: Arzneiverordnungsreport (drug prescription report regarding the GKV system of Germany)
AMR: Antimicrobial resistance
DDD: Defined daily dose
GKV: Gesetzliche Krankenversicherung (statutory health insurance)
LCL: Lower control limit
PACF: Partial autocorrelation function
RKI: Robert Koch Institute
SPSS: Statistical Product and Service Solutions (software for statistical analyses, IBM)
TOP10: Group of the 10 most prescribed antibacterial drugs (based on the year 2022)
UCL: Upper control limit
WIdO: Wissenschaftliches Institut der AOK (scientific institute of the statutory local health insurances)
