# Supplementary material for: Long-term forecast for antibacterial drug consumption in Germany using ARIMA models
Source: Naunyn Schmiedebergs Arch Pharmacol. 2025 Jan 4;398(6):7409–28. doi: 10.1007/s00210-024-03721-4 (PMC12125074; doi:10.1007/s00210-024-03721-4)

**Long-term forecast for antibacterial drug consumption in Germany using ARIMA models**

**Lilly Josephine Bindel and Roland Seifert**

Supplemental Results

**Antibacterial drugs with multiple suitable ARIMA-models: Amoxicillin, cefuroxime axetil, doxycycline, amoxicillin clavulanic acid and ciprofloxacin**

For amoxicillin, four models were considered, including ARIMA(**0,1,0**), ARIMA(**0,1,3**), ARIMA(**3,1,0**) and ARIMA(3,1,3). Fit metrics and curves can be found in Table S1 and Fig. 4. Although the additional lags in the ARIMA(**3,1,0**) and ARIMA(**3,1,3**) models were not strictly significant (Fig. S1), they were included for completeness. When evaluating the fit values, ARIMA(**0,1,0**) emerged as the preferred model, exhibiting the lowest BIC and lowest model complexity. The prediction of DDD-prescriptions for 2040 across the models is relatively consistent, with ARIMA(**0,1,0**) predicting the highest (**84.8 million DDD-prescriptions**) and the other models suggesting slightly lower values. The prediction range for ARIMA(**0,1,0**) is also narrower, with moderate upper control limit (**UCL**) and lower control limit (**LCL**) values, making its predictions more realistic compared to the more extreme ranges of the other models.

For cefuroxime axetil, the four models evaluated include ARIMA(**0,1,0**), ARIMA(**1,1,1**), ARIMA(**1,1,8**) and ARIMA(**1,1,11**). Fit metrics and curves can be found in Table S2 and Fig. S11. While the fit values for the significant (Fig. S2), but more complex ARIMA(**1,1,8**) and ARIMA(**1,1,11**) models show better stationary R-squared values, ARIMA(**0,1,0**) provides the lowest BIC and thus the most parsimonious model. ARIMA(**1,1,11**) had the highest R-squared and Stationary R-squared values, making it perform better in terms of capturing the data’s variability. However, ARIMA(**0,1,0**) predicted a lower and more realistic increase in DDD-prescriptions by 2040, with the model predicting 54.1 DDD-prescriptions compared to 75.2 DDD-prescriptions in ARIMA(**1,1,8**). The narrow range and low BIC of ARIMA(**0,1,0**) make it the best fit for forecasting.

For doxycycline, two models were evaluated, including ARIMA(**0,1,0**) and ARIMA(**8,1,0**). Fit metrics and curves can be found in Table S3 and Fig. S12. The significant (Fig. S3) ARIMA(**8,1,0**) model has marginally better R-squared values, but ARIMA(**0,1,0**) was preferred due to its lower BIC and simpler structure. The predictions for 2040 were similar across both models, with ARIMA(**0,1,0**) predicting slightly higher DDD-prescriptions (**29.5 million**) compared to ARIMA(**8,1,0**) (**29.1 million**). The range of predictions was also tighter for ARIMA(**0,1,0**), making it the more reliable model for this antibacterial drug.

For amoxicillin clavulanic acid, the two models compared were ARIMA(**0,1,0**) and ARIMA(**0,1,5**). Fit metrics and curves can be found in Table S4 and Fig. 5. Both models produced similar fit values, but ARIMA(**0,1,0**) demonstrated a slightly better BIC and lower complexity. The forecast for ARIMA(**0,1,5**) predicted a slightly higher DDD-prescription value for 2040, but ARIMA(**0,1,0**)’s narrower prediction range for the UCL and LCL rendered it the more realistic option. ARIMA(0,1,0) was thus deemed more suitable for prediction (Table S4, Fig. S4).

For ciprofloxacin, the models ARIMA(**0,1,0**) and ARIMA(**1,1,1**) were assessed. Fit metrics and curves can be found in Table S5 and Fig. S18. Although the significant (Fig. S10) ARIMA(**1,1,1**) showed better R-squared values, ARIMA(**0,1,0**) had a lower BIC, making it the preferred model. ARIMA(**1,1,1**) predicted a higher increase in DDD-prescriptions for 2040 and a much wider range of possible outcomes, with an unrealistically high UCL. Therefore, despite its slightly worse fit values, ARIMA(**0,1,0**) was selected due to its more conservative and realistic forecast.

Summarized, for each of the antibacterial drugs, ARIMA(**0,1,0**) generally proved to be the most robust model in terms of balancing model simplicity, low BIC and realistic prediction ranges. While more complex models sometimes yielded marginally better fit values, their predictions were often accompanied by wide and potentially unrealistic ranges.

Supplemental Tables

***Table S1:*** *Overview about parameters, fit values and predictions for possible ARIMA-models of the antibacterial drug amoxicillin. If a lag for the parameters is written in brackets, it is narrowly significant but did not fulfil the criteria, nevertheless, they are included so no information is missed. The table evaluates model fit based on several metrics. A good fit (green colour) is indicated by high Stationary R-squared (above 0.8), high R-squared (above 0.7), and low values for RMSE, MAPE, MaxAPE, MAE, MaxAE, and Normalized BIC. A medium fit (yellow colour) falls between 0.5-0.8 for Stationary R-squared, 0.4-0.7 for R-squared, and moderate values (10-20%) for MAPE, MaxAPE, and MaxAE. A poor fit (orange colour) is characterized by low R-squared (below 0.4), high errors (MAPE, MaxAPE, and MaxAE above 20%), and the highest RMSE, MAE, and BIC values (Hyndman and Athanasopoulos 2018).* *Variants where the AR or MA components exceed zero include all respective lags. For example, ARIMA(0,1,3) incorporates one level of differencing along with all three moving average terms: MA1, MA2, and MA3.*

| **antibacterial drug** | **1 Amoxicillin** | | | |
| --- | --- | --- | --- | --- |
| significant lags in PAFC (p) | 0, (3) | | | |
| degree of differentiation (d) | 1 | | | |
| significant lags in ACF (q) | 0, (3) | | | |
| model | ARIMA(0,1,0) | ARIMA(0,1,3) | ARIMA(3,1,0) | ARIMA(3,1,3) |
| Stationary R-squared | -2.22045E-16 | 0.09638506 | 0.12164315 | 0.1931973 |
| R-squared | 0.903670863 | 0.91295555 | 0.91538864 | 0.92228139 |
| RMSE | 8.134240433 | 8.07612416 | 7.96245118 | 8.00370919 |
| MAPE | 10.86514237 | 10.9533545 | 11.1250803 | 10.7690138 |
| MaxAPE | 43.53192972 | 42.6078636 | 43.5504224 | 45.5672282 |
| MAE | 5.265113221 | 5.36919611 | 5.44244694 | 5.27710993 |
| MaxAE | 26.06216216 | 24.5243351 | 23.6517972 | 24.7430049 |
| Normalized BIC | 4.289757108 | 4.56819365 | 4.53984328 | 4.8429568 |
| trend | increasing | increasing | increasing | increasing |
| outliers | no | no | no | no |
| possibility for decrease | yes | yes | yes | yes |
| DDD-prescriptions in 2022 | 58.5 | | | |
| predicted DDD-prescriptions for 2040 | 84.8 | 83.8 | 76.6 | 80.1 |
| relative change 2040 to 2022 in % | + 45.0% | + 43.2% | + 30.9 % | + 36.9 % |
| predicted UCL for 2040 | 154.8 | 169.3 | 160.7 | 156.6 |
| relative change 2040 to 2022 in % | +164.6 % | + 189.4 % | + 174.7 % | + 167.7 % |
| predicted LCL for 2040 | 14.8 | 0 | 0 | 3.6 |
| relative change 2040 to 2022 in % | -74.7 % | - 100.0% | - 100.0% | - 93.8 % |

***Table S2:*** *Overview about parameters, fit values and predictions for possible ARIMA-models of the antibacterial drug cefuroxime axetil. The table evaluates model fit based on several metrics. A good fit (green colour) is indicated by high Stationary R-squared (above 0.8), high R-squared (above 0.7), and low values for RMSE, MAPE, MaxAPE, MAE, MaxAE, and Normalized BIC. A medium fit (yellow colour) falls between 0.5-0.8 for Stationary R-squared, 0.4-0.7 for R-squared, and moderate values (10-20%) for MAPE, MaxAPE, and MaxAE. A poor fit (orange colour) is characterized by low R-squared (below 0.4), high errors (MAPE, MaxAPE, and MaxAE above 20%), and the highest RMSE, MAE, and BIC values (Hyndman and Athanasopoulos 2018). Variants where the AR or MA components are greater than zero include all corresponding lags. For instance, ARIMA(1,1,8) incorporates one level of differencing, the first autoregressive term (AR1), and all eight moving average terms (MA1, MA2, MA3, MA4, MA5, MA6, MA7, MA8).*

| **antibacterial drug** | **2 Cefuroxime axetil** | | | |
| --- | --- | --- | --- | --- |
| significant lags in PAFC (p) | 0, 1 | | | |
| degree of differentiation (d) | 1 | | | |
| significant lags in ACF (q) | 0, 1, 8, 11 | | | |
| model | ARIMA(0,1,0) | ARIMA(1,1,1) | ARIMA(1,1,8) | ARIMA(1,1,11) |
| Stationary R-squared | 1.11022E-16 | 0.21963969 | 0.410693492 | 0.57159695 |
| R-squared | 0.943572847 | 0.95596649 | 0.966747111 | 0.97582644 |
| RMSE | 4.715997782 | 4.30727923 | 4.297478547 | 3.94279245 |
| MAPE | 16.51431905 | 12.4484169 | 13.24294668 | 11.3105568 |
| MaxAPE | 45.73518237 | 40.777252 | 35.15012596 | 41.3211578 |
| MAE | 3.337304688 | 2.65113578 | 2.567199008 | 2.23905452 |
| MaxAE | 15.046875 | 13.4157159 | 9.897538176 | 7.29508589 |
| Normalized BIC | 3.210225272 | 3.24552561 | 3.999099402 | 4.15173364 |
| trend | increasing | increasing | increasing | increasing |
| outliers | no | no | no | no |
| possibility for decrease | yes | yes | no | no |
| DDD-prescriptions in 2022 | 35.3 | | | |
| predicted DDD-prescriptions for 2040 | 54.1 | 63.7 | 75.2 | 74.9 |
| relative change 2040 to 2022 in % | + 52.4 % | + 79.4 % | + 111.8 % | + 110.9 % |
| predicted UCL for 2040 | 95 | 133.1 | 108.4 | 120.5 |
| relative change 2040 to 2022 in % | + 169.1 % | + 274.9 % | + 207.1 % | + 240.0 % |
| predicted LCL for 2040 | 13.3 | 0 | 42.0 | 29.2 |
| relative change 2040 to 2022 in % | - 62.3 % | - 100.0% | +18.9 % | -17.3 % |

***Table S3:*** *Overview about parameters, fit values and predictions for possible ARIMA-models of the antibacterial drug doxycycline. If a lag for the parameters is written in brackets, it is narrowly significant but did not fulfil the criteria, nevertheless, they are included so no information is missed. The table evaluates model fit based on several metrics. A good fit (green colour) is indicated by high Stationary R-squared (above 0.8), high R-squared (above 0.7), and low values for RMSE, MAPE, MaxAPE, MAE, MaxAE, and Normalized BIC. A medium fit (yellow colour) falls between 0.5-0.8 for Stationary R-squared, 0.4-0.7 for R-squared, and moderate values (10-20%) for MAPE, MaxAPE, and MaxAE. A poor fit (orange colour) is characterized by low R-squared (below 0.4), high errors (MAPE, MaxAPE, and MaxAE above 20%), and the highest RMSE, MAE, and BIC values (Hyndman and Athanasopoulos 2018). Variants where the AR or MA components are greater than zero include all corresponding lags. For example, ARIMA(8,1,0) incorporates one level of differencing and all eight autoregressive terms (AR1, AR2, AR3, AR4, AR5, AR6, AR7, AR8).*

| **antibacterial drug** | **3 Doxycycline** | |
| --- | --- | --- |
| significant lags in PAFC (p) | 0, (8) | |
| degree of differentiation (d) | 1 | |
| significant lags in ACF (q) | 0 | |
| model | ARIMA(0,1,0) | ARIMA(8,1,0) |
| Stationary R-squared | 0 | 0.23865021 |
| R-squared | 0.80437794 | 0.85106318 |
| RMSE | 5.49586386 | 5.43751309 |
| MAPE | 7.04027376 | 6.42181495 |
| MaxAPE | 19.9077901 | 16.4807953 |
| MAE | 4.08239591 | 3.69228311 |
| MaxAE | 16.9216216 | 13.9213309 |
| Normalized BIC | 3.50558394 | 4.26497499 |
| trend | decreasing | decreasing |
| outliers | no | no |
| possibility for decrease | yes | yes |
| DDD-prescriptions in 2022 | 33.5 | |
| predicted DDD-prescriptions for 2040 | 29.5 | 29.1 |
| relative change 2040 to 2022 in % | - 11.9 % | - 13.1 % |
| predicted UCL for 2040 | 76.8 | 85.7 |
| relative change 2040 to 2022 in % | + 129.2 % | + 155.8 % |
| predicted LCL for 2040 | 0 | 0 |
| relative change 2040 to 2022 in % | - 100.0 % | -100.0 % |

***Table S4:*** *Overview about parameters, fit values and predictions for possible ARIMA-models of the antibacterial drug amoxicillin clavulanic acid. If a lag for the parameters is written in brackets, it is narrowly significant but did not fulfil the criteria, nevertheless, they are included so no information is missed. The table evaluates model fit based on several metrics. A good fit (green colour) is indicated by high Stationary R-squared (above 0.8), high R-squared (above 0.7), and low values for RMSE, MAPE, MaxAPE, MAE, MaxAE, and Normalized BIC. A medium fit (yellow colour) falls between 0.5-0.8 for Stationary R-squared, 0.4-0.7 for R-squared, and moderate values (10-20%) for MAPE, MaxAPE, and MaxAE. A poor fit (orange colour) is characterized by low R-squared (below 0.4), high errors (MAPE, MaxAPE, and MaxAE above 20%), and the highest RMSE, MAE, and BIC values (Hyndman and Athanasopoulos 2018). Variants where the AR or MA components are greater than zero include all corresponding lags. For instance, ARIMA(0,1,5) incorporates one level of differencing and all five moving average terms (MA1, MA2, MA3, MA4, MA5).*

| **antibacterial drug** | **4 Amoxicillin clavulanic acid** | |
| --- | --- | --- |
| significant lags in PAFC (p) | 0 | |
| degree of differentiation (d) | 1 | |
| significant lags in ACF (q) | 0, (5) | |
| model | ARIMA(0,1,0) | ARIMA(0,1,5) |
| Stationary R-squared | 2.2204E-16 | 0.32176876 |
| R-squared | 0.9483086 | 0.96494128 |
| RMSE | 1.52306083 | 1.35481357 |
| MAPE | 31.1720399 | 32.7756717 |
| MaxAPE | 212.5 | 230.586049 |
| MAE | 0.875 | 0.8523364 |
| MaxAE | 6.95 | 4.04662562 |
| Normalized BIC | 0.94098622 | 1.20458087 |
| trend | increasing | increasing |
| outliers | no | no |
| possibility for decrease | no | no |
| DDD-prescriptions in 2022 | 27.5 | |
| predicted DDD-prescriptions for 2040 | 41 | 45.3 |
| relative change 2040 to 2022 in % | + 49.1 % | + 64.7 % |
| predicted UCL for 2040 | 54.1 | 62.6 |
| relative change 2040 to 2022 in % | + 96.7 % | + 127.6 % |
| predicted LCL for 2040 | 27.9 | 28.0 |
| relative change 2040 to 2022 in % | + 0.7 % | + 1.8 % |

***Table S5:*** *Overview about parameters, fit values and predictions for possible ARIMA-models of the antibacterial drug ciprofloxacin. If a lag for the parameters is written in brackets, it is narrowly significant but did not fulfil the criteria, nevertheless, they are included so no information is missed. The table evaluates model fit based on several metrics. A good fit (green colour) is indicated by high Stationary R-squared (above 0.8), high R-squared (above 0.7), and low values for RMSE, MAPE, MaxAPE, MAE, MaxAE, and Normalized BIC. A medium fit (yellow colour) falls between 0.5-0.8 for Stationary R-squared, 0.4-0.7 for R-squared, and moderate values (10-20%) for MAPE, MaxAPE, and MaxAE. A poor fit (orange colour) is characterized by low R-squared (below 0.4), high errors (MAPE, MaxAPE, and MaxAE above 20%), and the highest RMSE, MAE, and BIC values (Hyndman and Athanasopoulos 2018).*

| **antibacterial drug** | **10** **Ciprofloxacin** | |
| --- | --- | --- |
| significant lags in PAFC (p) | 1 | |
| degree of differentiation (d) | 1 | |
| significant lags in ACF (q) | 1 | |
| model | ARIMA(0,1,0) | ARIMA(1,1,1) |
| Stationary R-squared | 4.4409E-16 | 0.15574341 |
| R-squared | 0.87672532 | 0.89592454 |
| RMSE | 1.96108356 | 1.85736731 |
| MAPE | 16.1602422 | 14.607788 |
| MaxAPE | 122.315271 | 127.538185 |
| MAE | 1.33257143 | 1.1519827 |
| MaxAE | 7.09428571 | 7.39721471 |
| Normalized BIC | 1.44857569 | 1.54306424 |
| trend | increasing | increasing |
| outliers | no | no |
| possibility for decrease | yes | yes |
| DDD in 2022 | 7.7 | |
| predicted DDD for 2040 | 11.2 | 11.7 |
| relative change 2040 to 2022 in % | + 45.5 % | + 51.9 % |
| predicted UCL for 2040 | 28.1 | 39.4 |
| relative change 2040 to 2022 in % | + 264.9 % | + 411.7 % |
| predicted LCL for 2040 | 0 | 0 |
| relative change 2040 to 2022 in % | - 100.0 % | - 100.0 % |

***Table S6:*** *Predictions for future DDD-prescriptions for the ten most prescribed antibacterial drugs for the next 5 years (2027) and 10 years (2032). The prediction is based on the ARIMA(0,1,0) model. If other ARIMA models are considered as possible, their forecast is quite similar.*

| **antibacterial drug** | **DDD-prescriptions in million in 2022** | **predicted DDD-prescriptions in million in 2027** | **predicted DDD-prescriptions in million in 2032** |
| --- | --- | --- | --- |
| 1 Amoxicillin | 58.5 | 65.8 | 73.1 |
| 2 Cefuroxime axetil | 35.3 | 40.5 | 45.8 |
| 3 Doxycycline | 33.5 | 32.4 | 31.3 |
| 4 Amoxicillin clavulanic acid | 27.5 | 31.3 | 35.0 |
| 5 Clindamycin | 13.5 | 15.1 | 15.4 |
| 6 Azithromycin | 13.5 | 15.8 | 18.0 |
| 7 Phenoxymethylpenicillin | 10.8 | 9.0 | 7.2 |
| 8 Sulfamethoxazole-Trimethoprim | 10.8 | 8.8 | 6.7 |
| 9 Nitrofurantoin | 9.1 | 9.5 | 9.8 |
| 10 Ciprofloxacin | 7.7 | 8.7 | 9.6 |

Supplemental Figures

***Fig. S1****: Autocorrelation parameters ACF and PACF of the original time series, before performing any differentiation, for amoxicillin.*


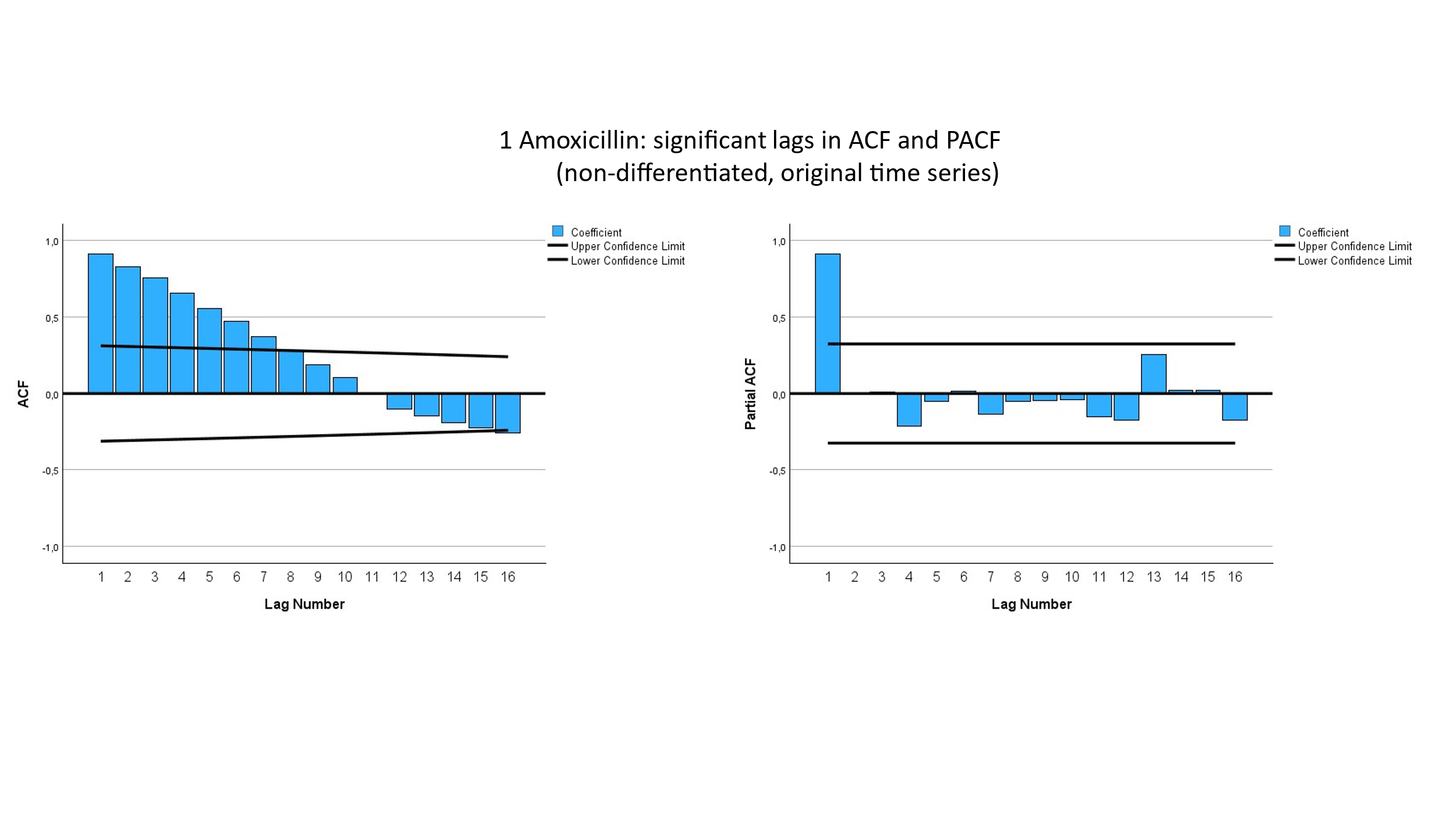


***Fig. S2****: Autocorrelation parameters ACF and PACF after performing one differentiation for amoxicillin. Significant lags, characterised by exceeding the black line, are considered as possible values for the respective ARIMA-model.*


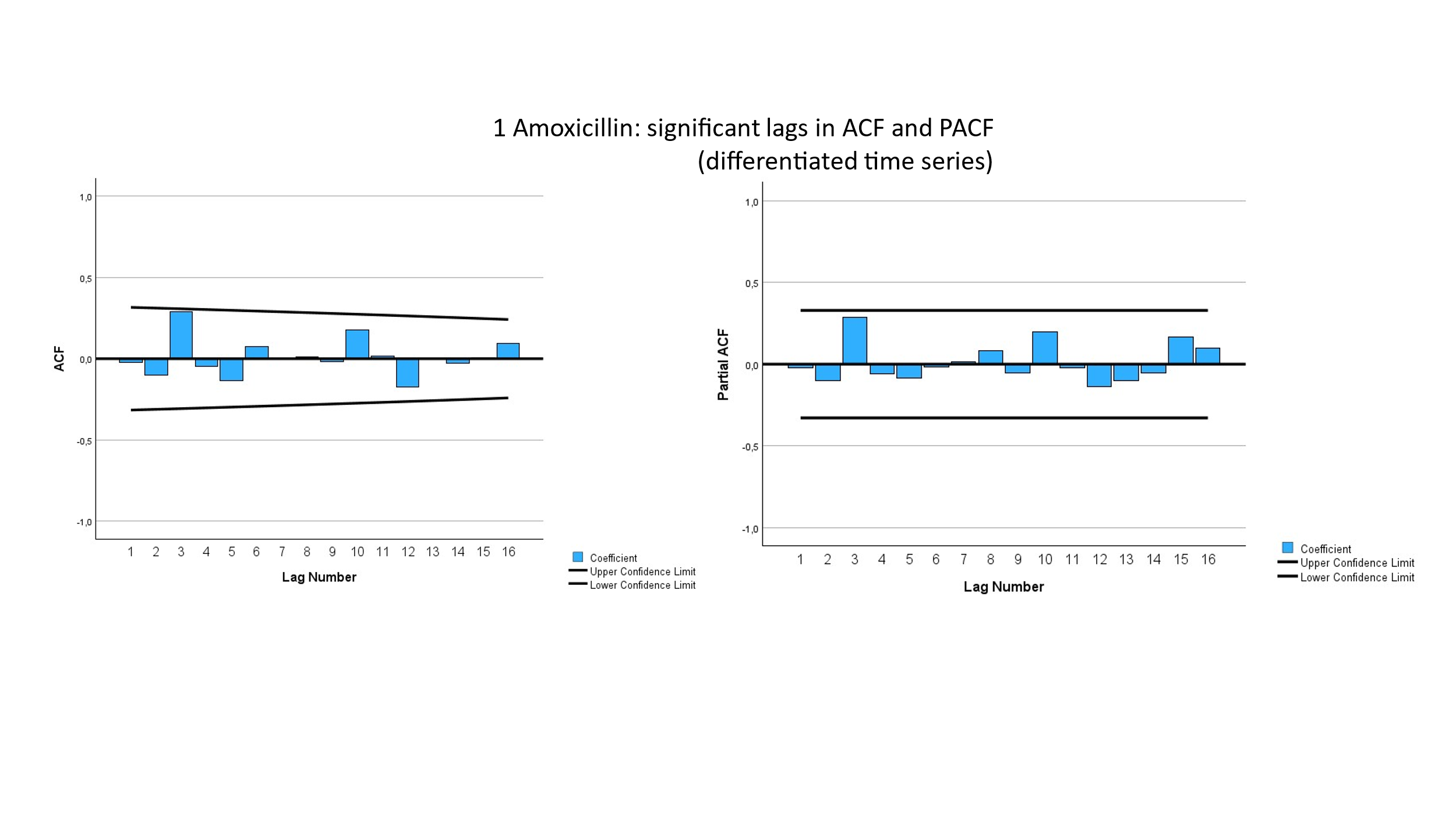


***Fig. S3****: Autocorrelation parameters ACF and PACF of the original time series, before performing any differentiation, for cefuroxime axetil.*


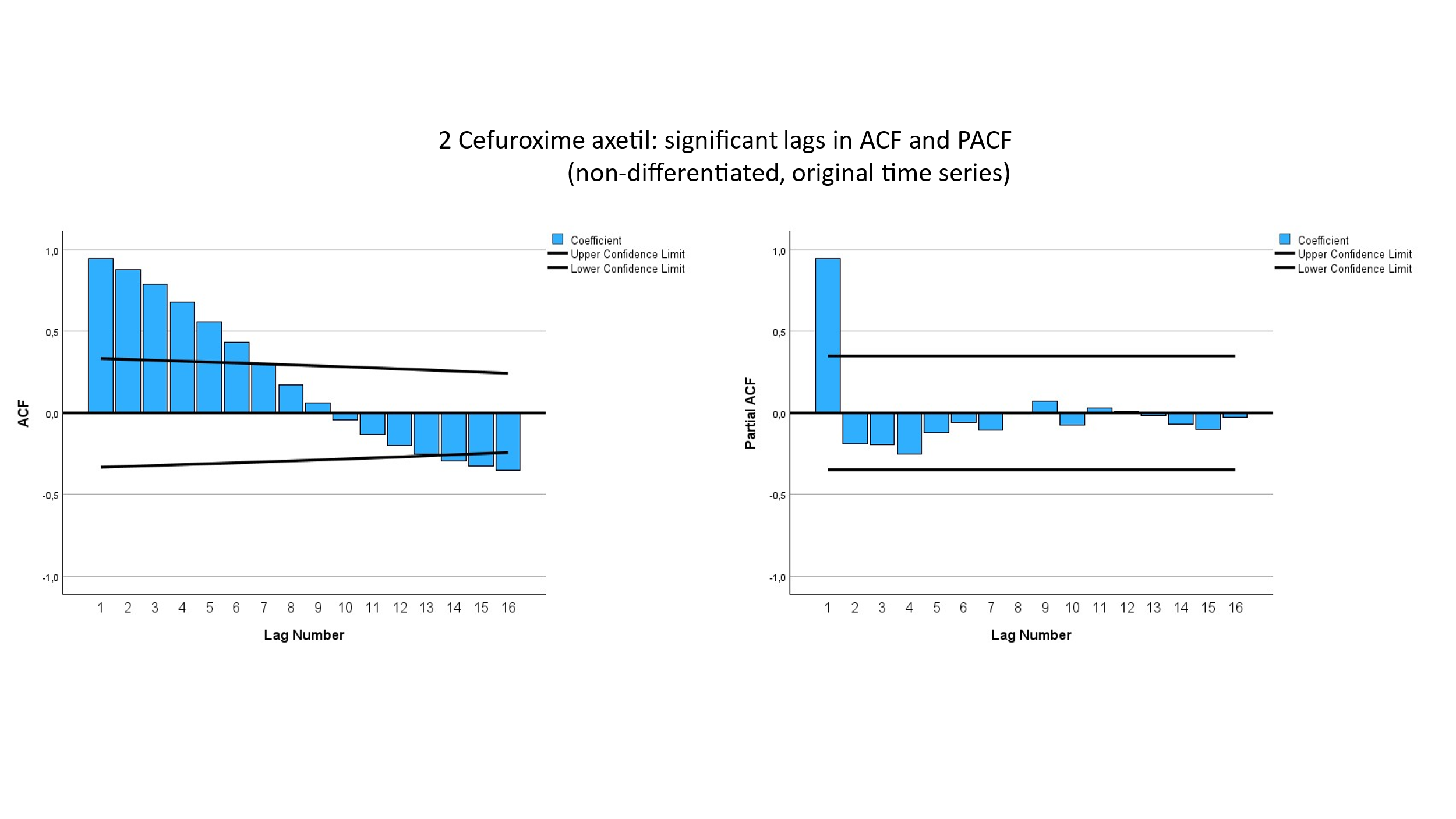


***Fig. S4****: Autocorrelation parameters ACF and PACF after performing one differentiation for cefuroxime axetil. Significant lags, characterised by exceeding the black line, are considered as possible values for the respective ARIMA-model.*


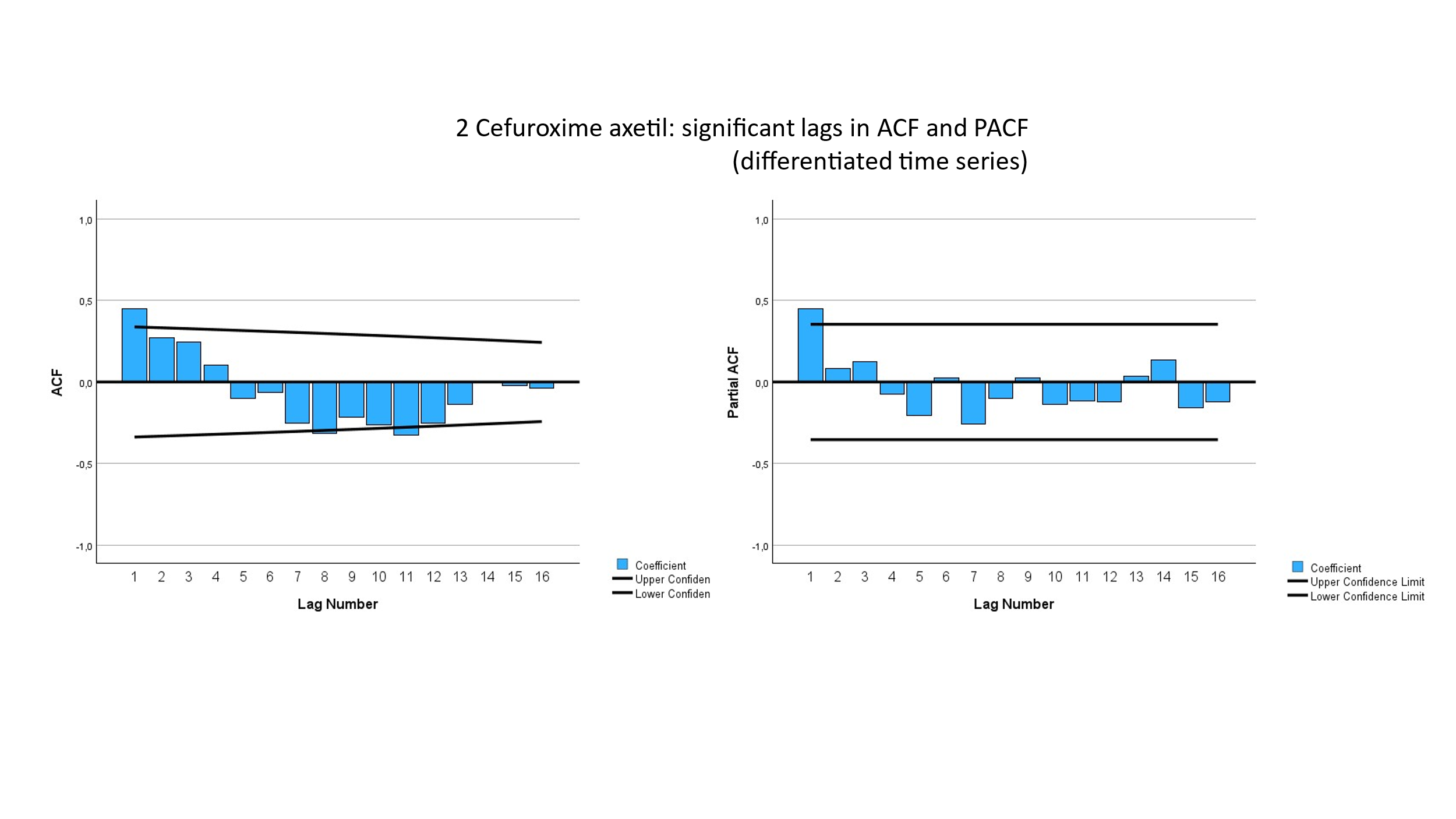


***Fig. S5****: Autocorrelation parameters ACF and PACF of the original time series, before performing any differentiation, for doxycycline.*

**
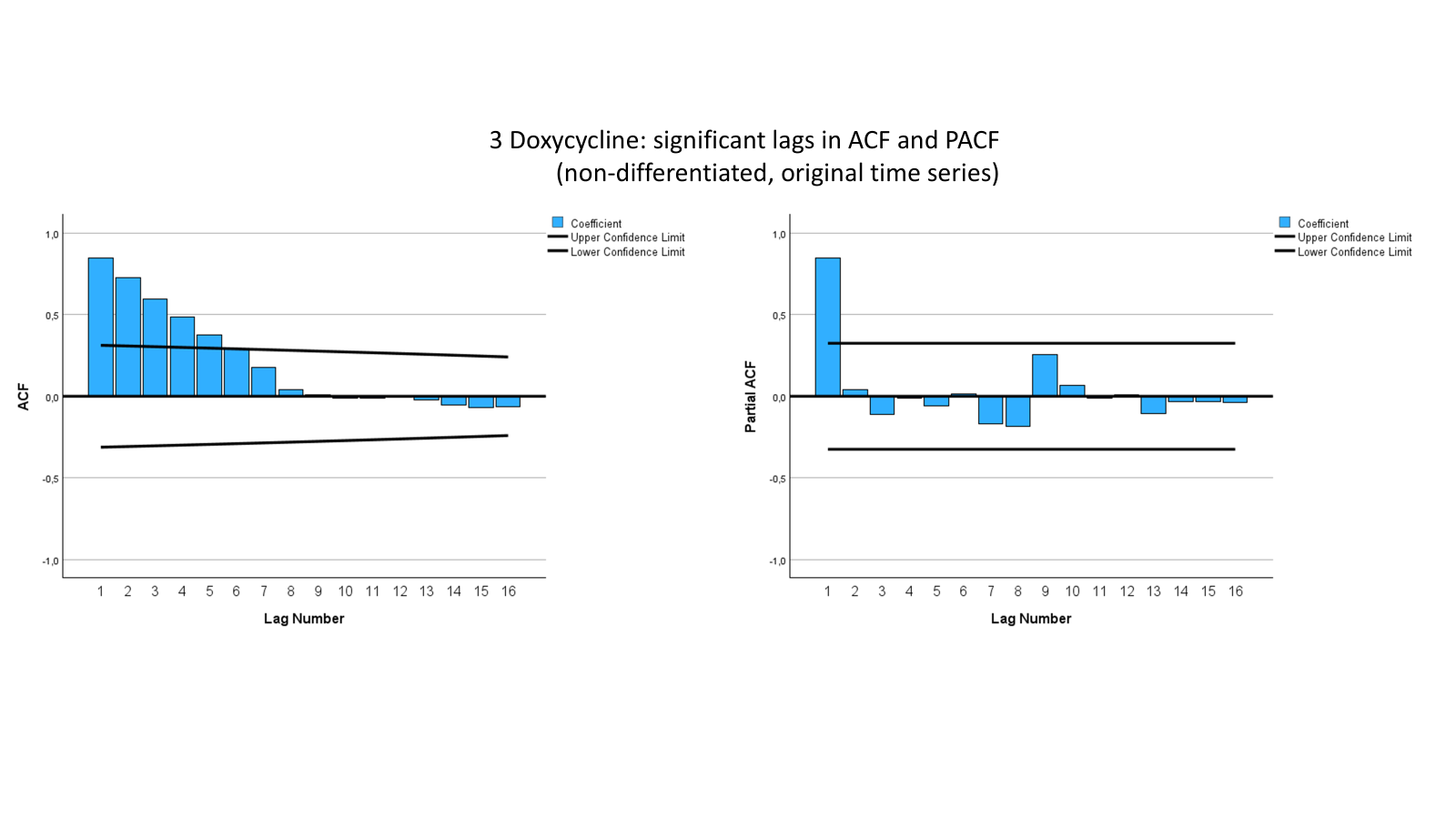
**

***Fig. S6****: Autocorrelation parameters ACF and PACF after performing one differentiation for doxycycline. Significant lags, characterised by exceeding the black line, are considered as possible values for the respective ARIMA-model.*

*
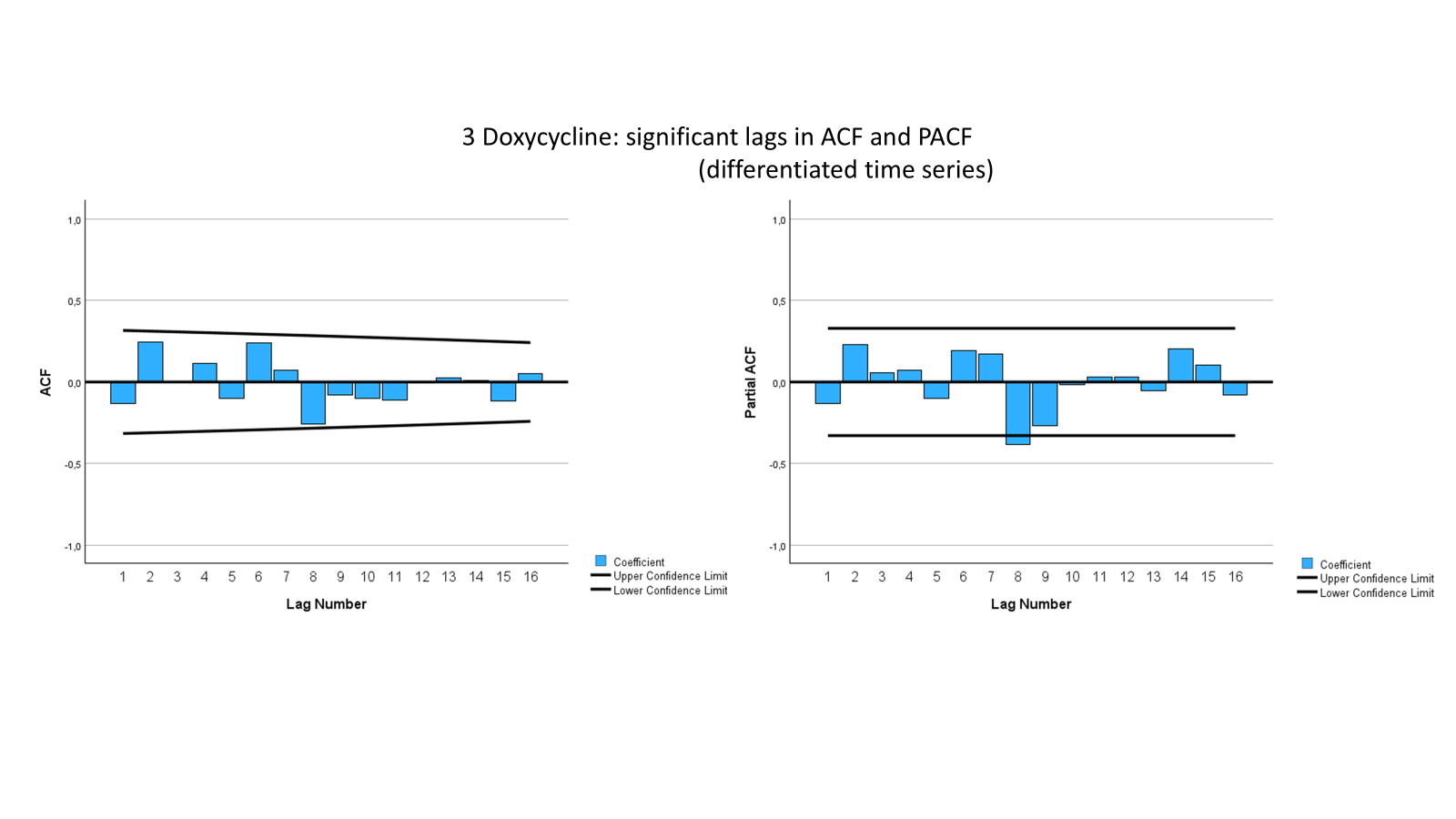
*

***Fig. S7****: Autocorrelation parameters ACF and PACF of the original time series, before performing any differentiation, for amoxicillin clavulanic acid.*

**
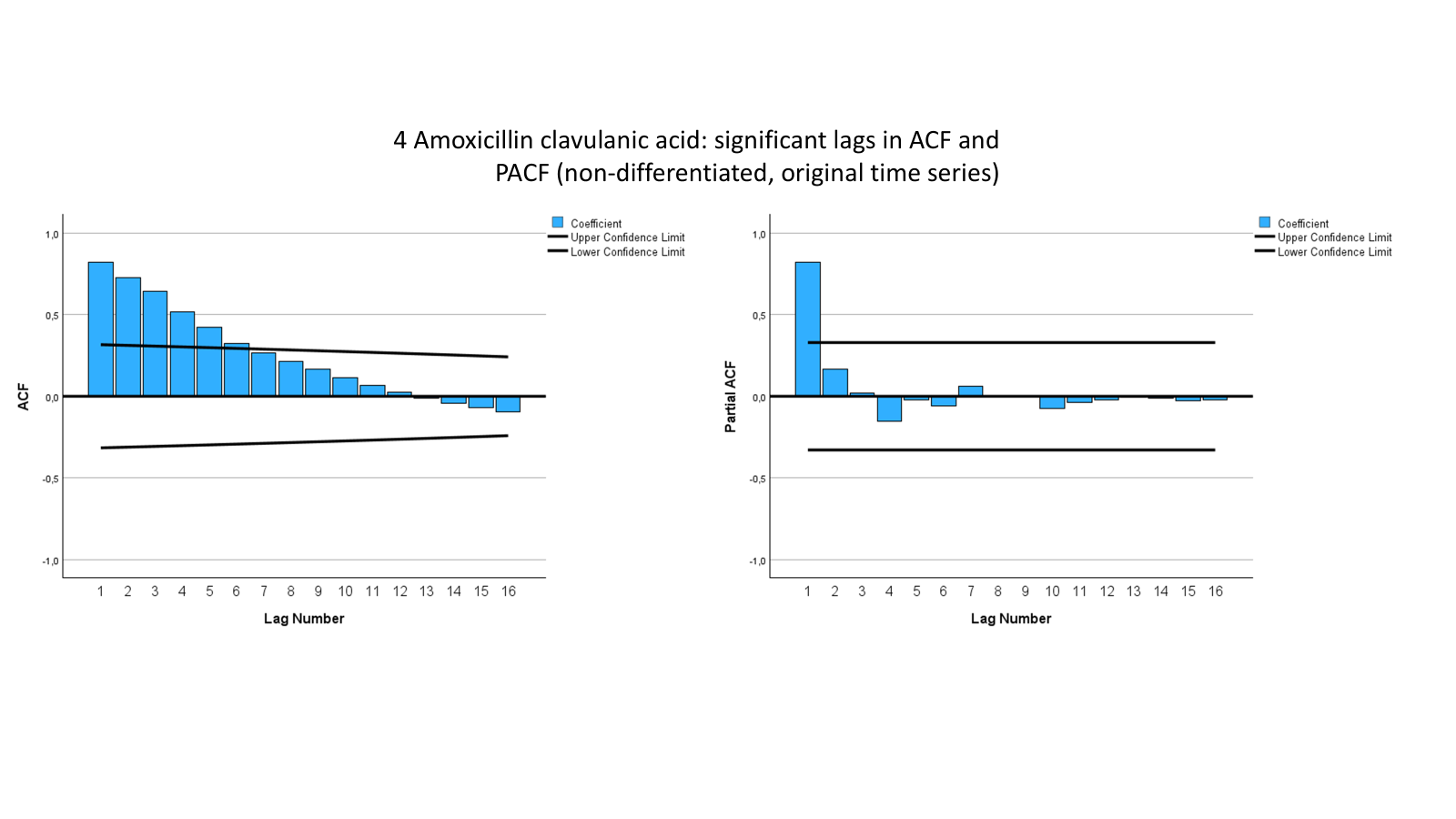
**

***Fig. S8****: Autocorrelation parameters ACF and PACF after performing one differentiation for amoxicillin clavulanic acid. Significant lags, characterised by exceeding the black line, are considered as possible values for the respective ARIMA-model.*


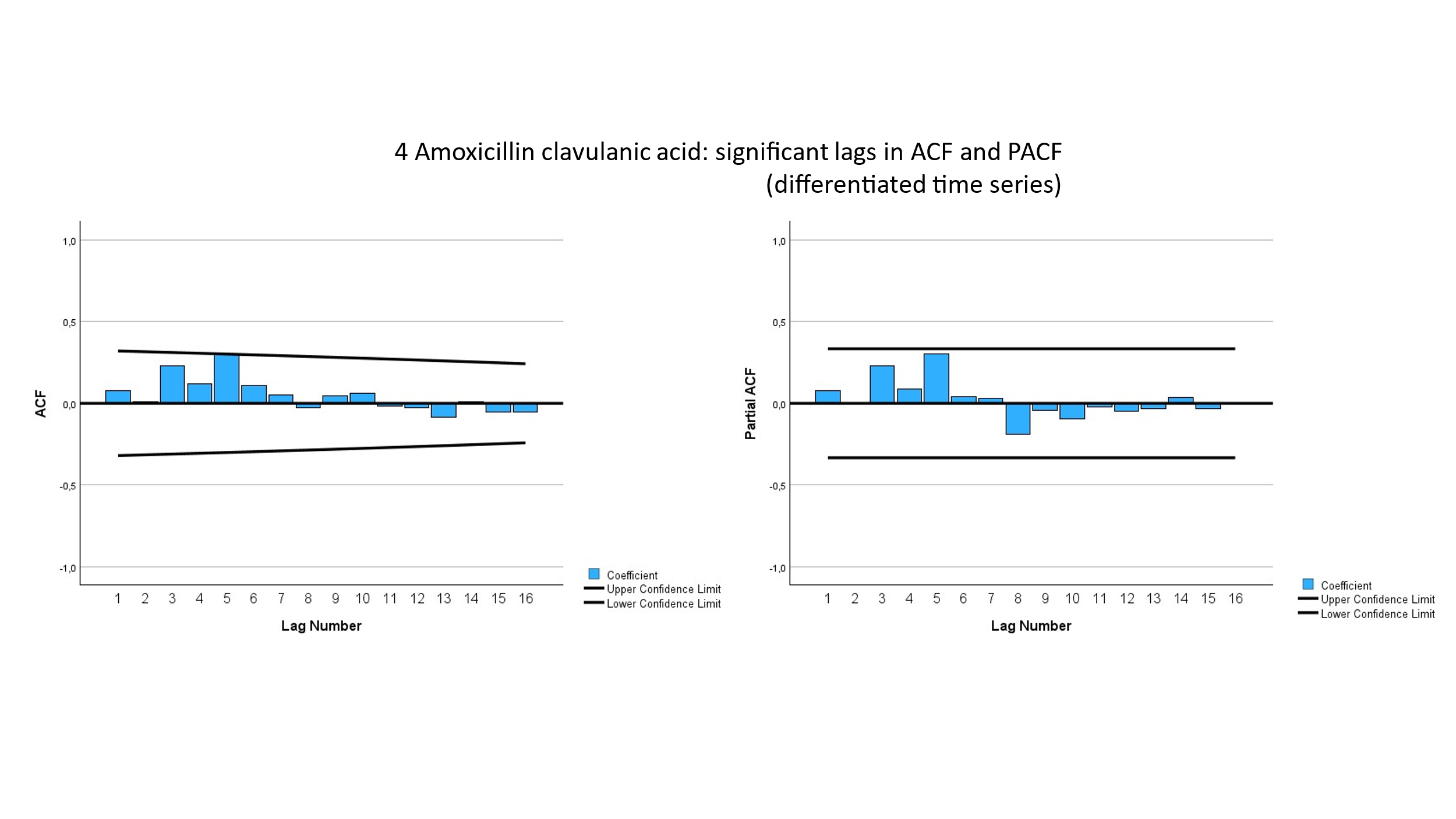


***Fig. S9****: Autocorrelation parameters ACF and PACF of the original time series, before performing any differentiation, for clindamycin.*

**
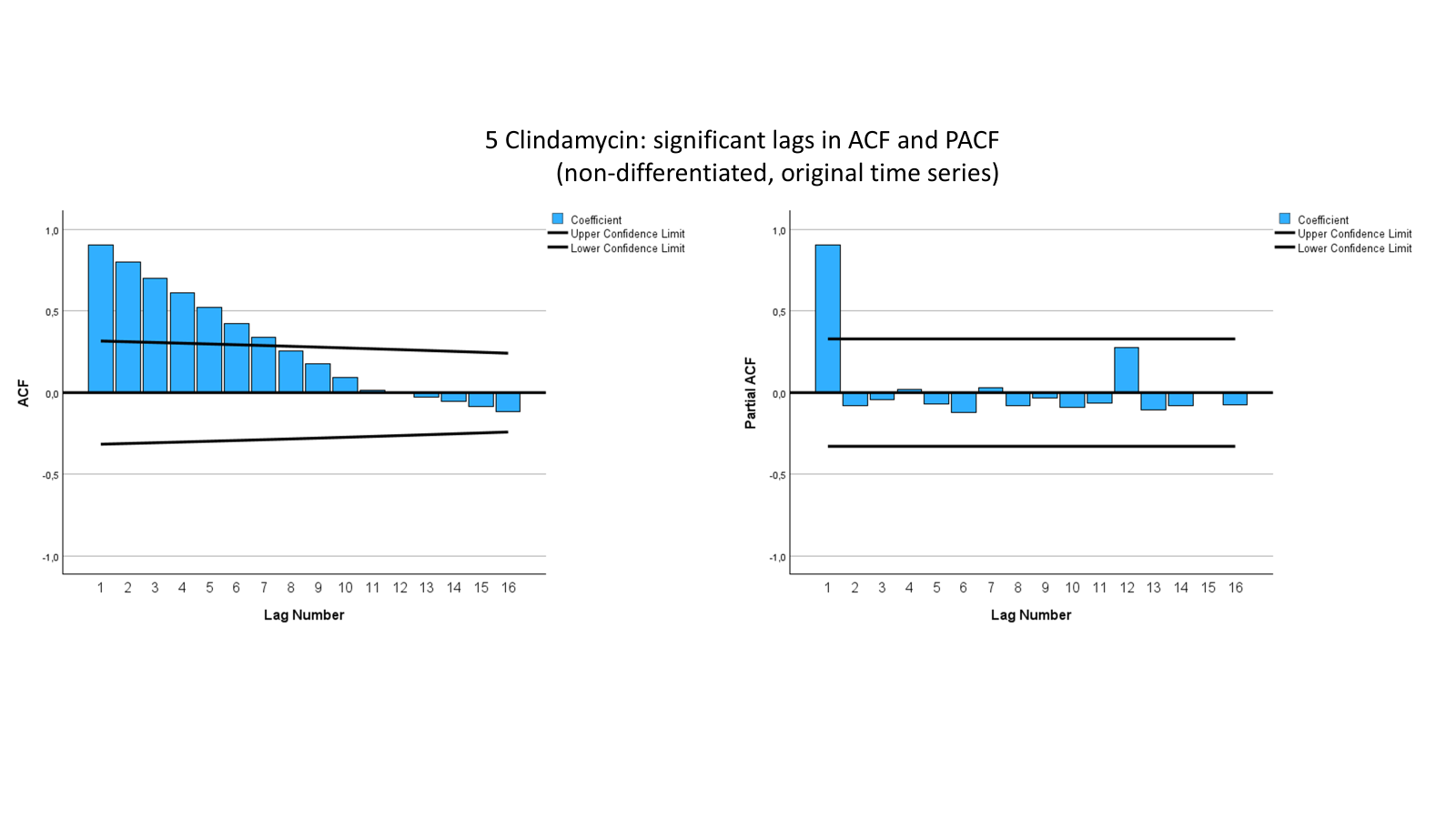
**

***Fig. S10****: Autocorrelation parameters ACF and PACF after performing one differentiation for clindamycin. Significant lags, characterised by exceeding the black line, are considered as possible values for the respective ARIMA-model.*


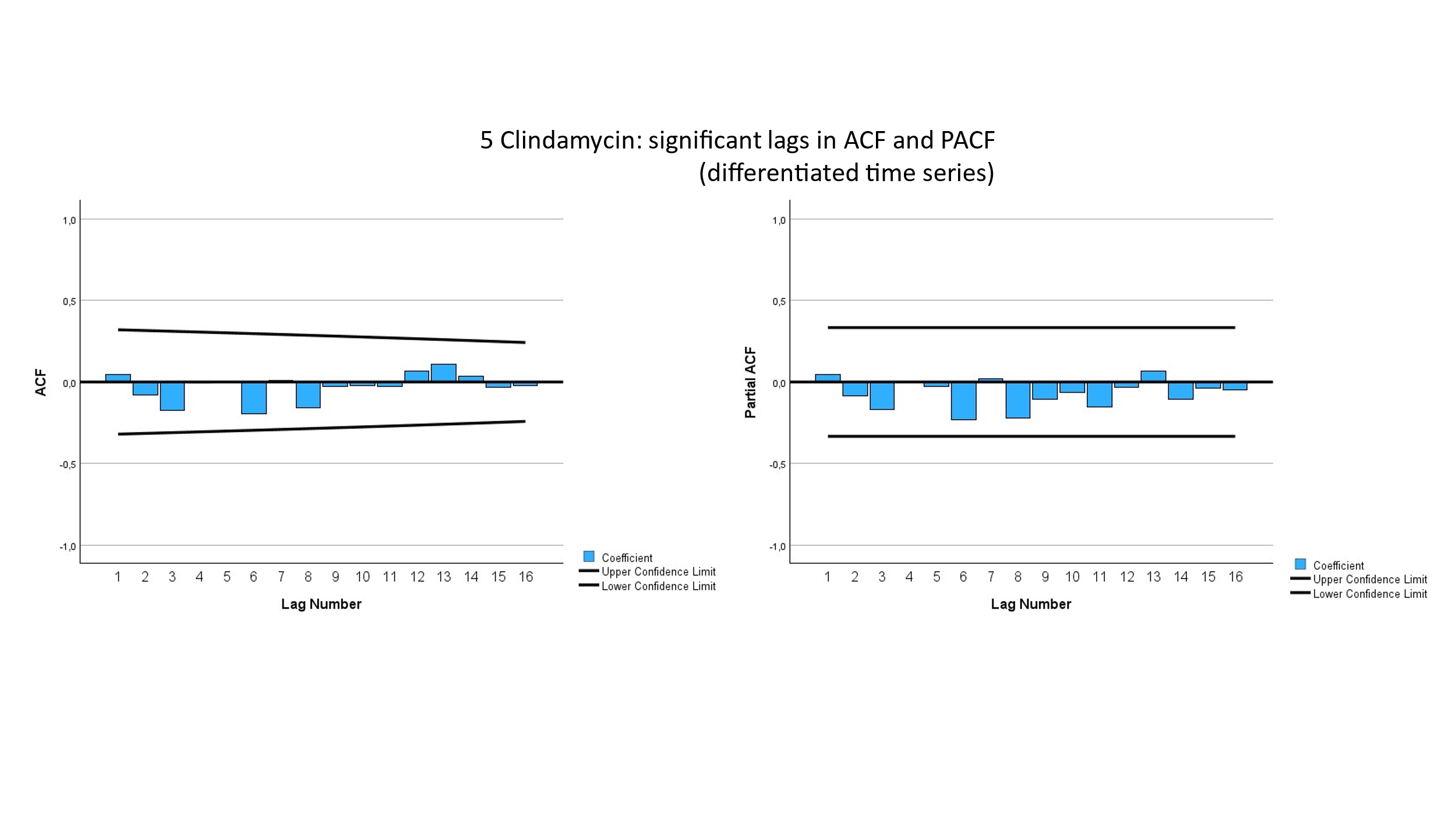


***Fig. S11****: Autocorrelation parameters ACF and PACF of the original time series, before performing any differentiation, for azithromycin.*

**
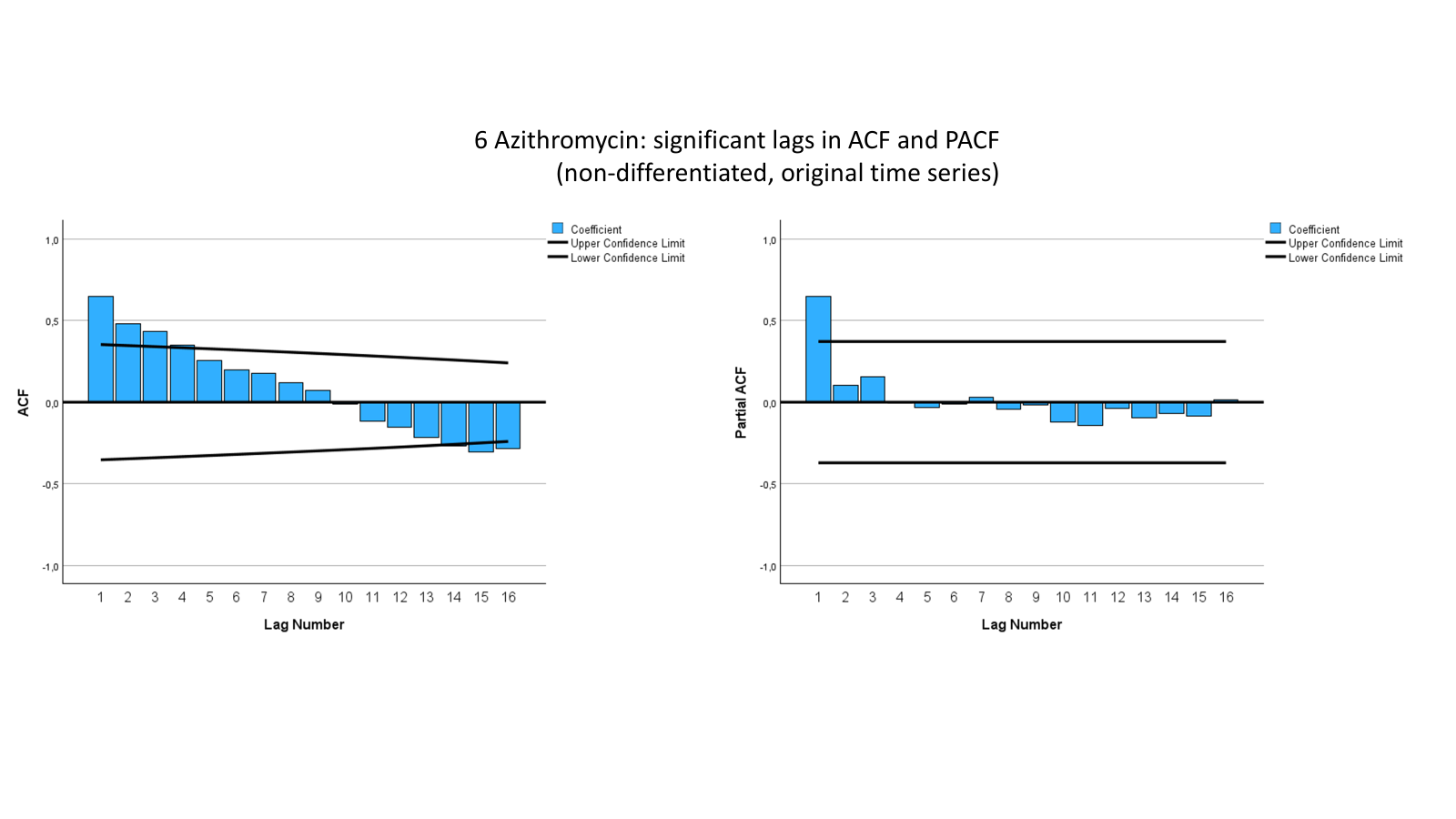
**

***Fig. S12****: Autocorrelation parameters ACF and PACF after performing one differentiation for azithromycin. Significant lags, characterised by exceeding the black line, are considered as possible values for the respective ARIMA-model.*


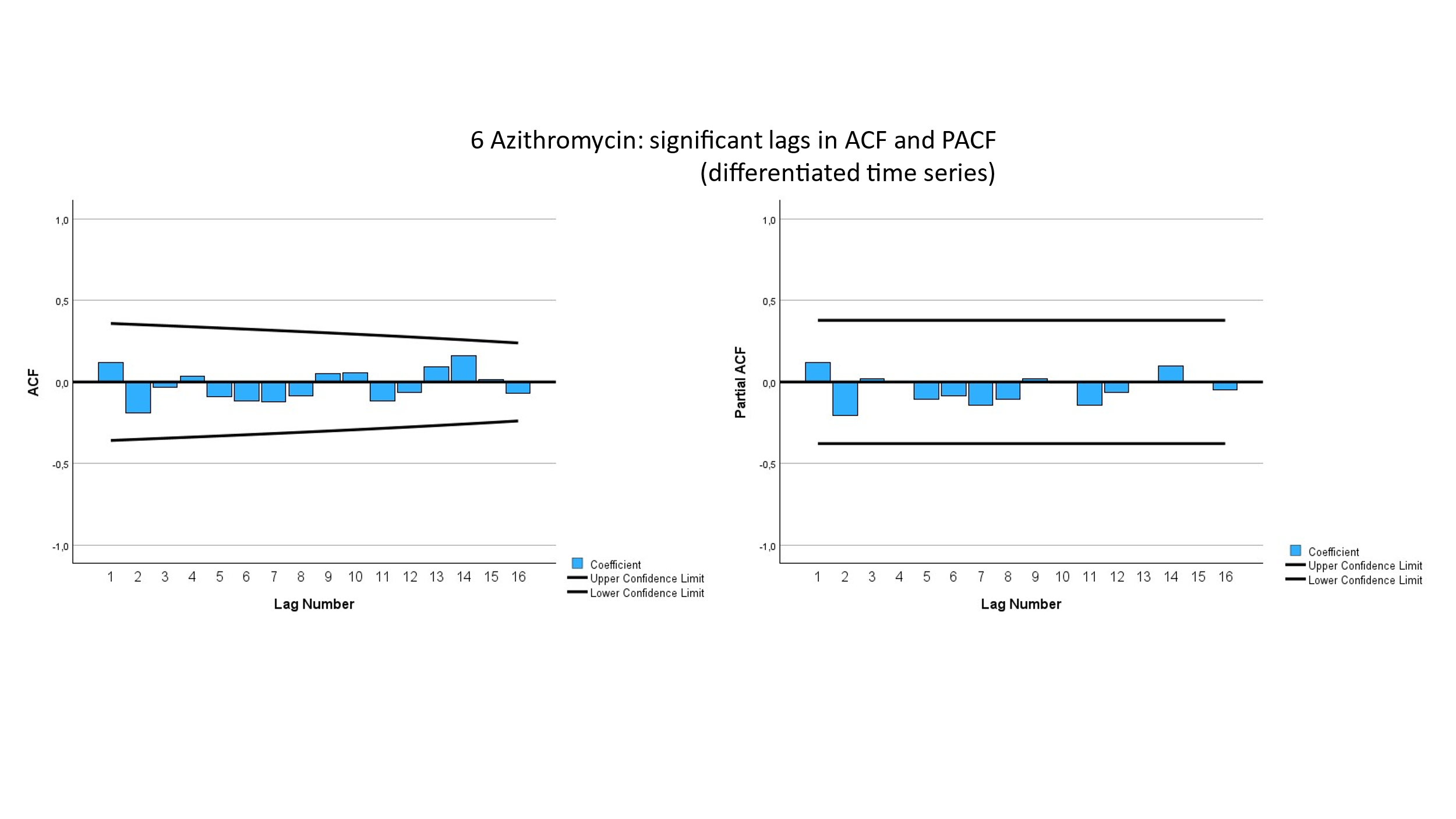


***Fig. S13****: Autocorrelation parameters ACF and PACF of the original time series, before performing any differentiation, for phenoxymethylpenicillin.*


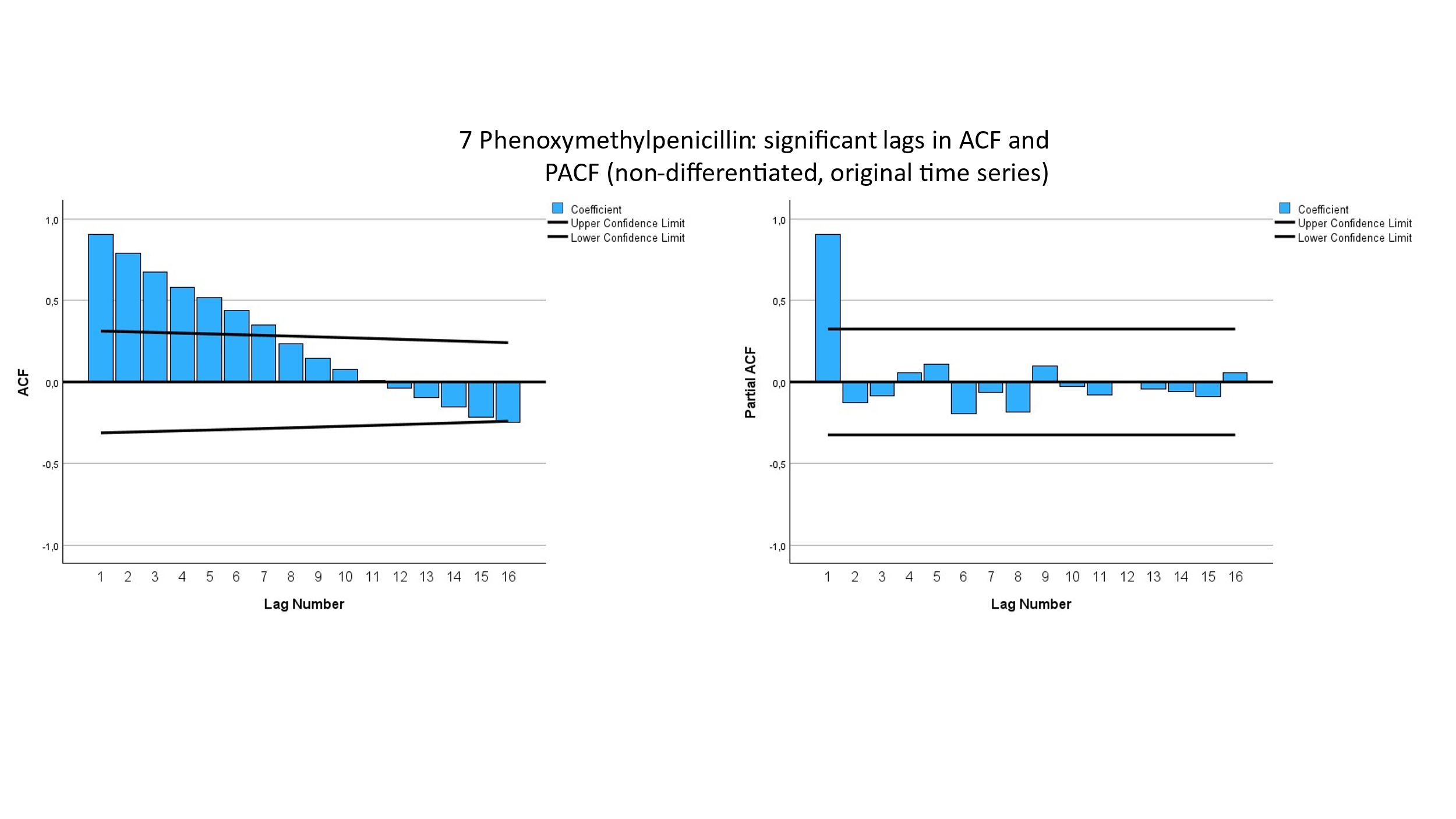


***Fig. S14****: Autocorrelation parameters ACF and PACF after performing one differentiation for phenoxymethylpenicillin. Significant lags, characterised by exceeding the black line, are considered as possible values for the respective ARIMA-model.*


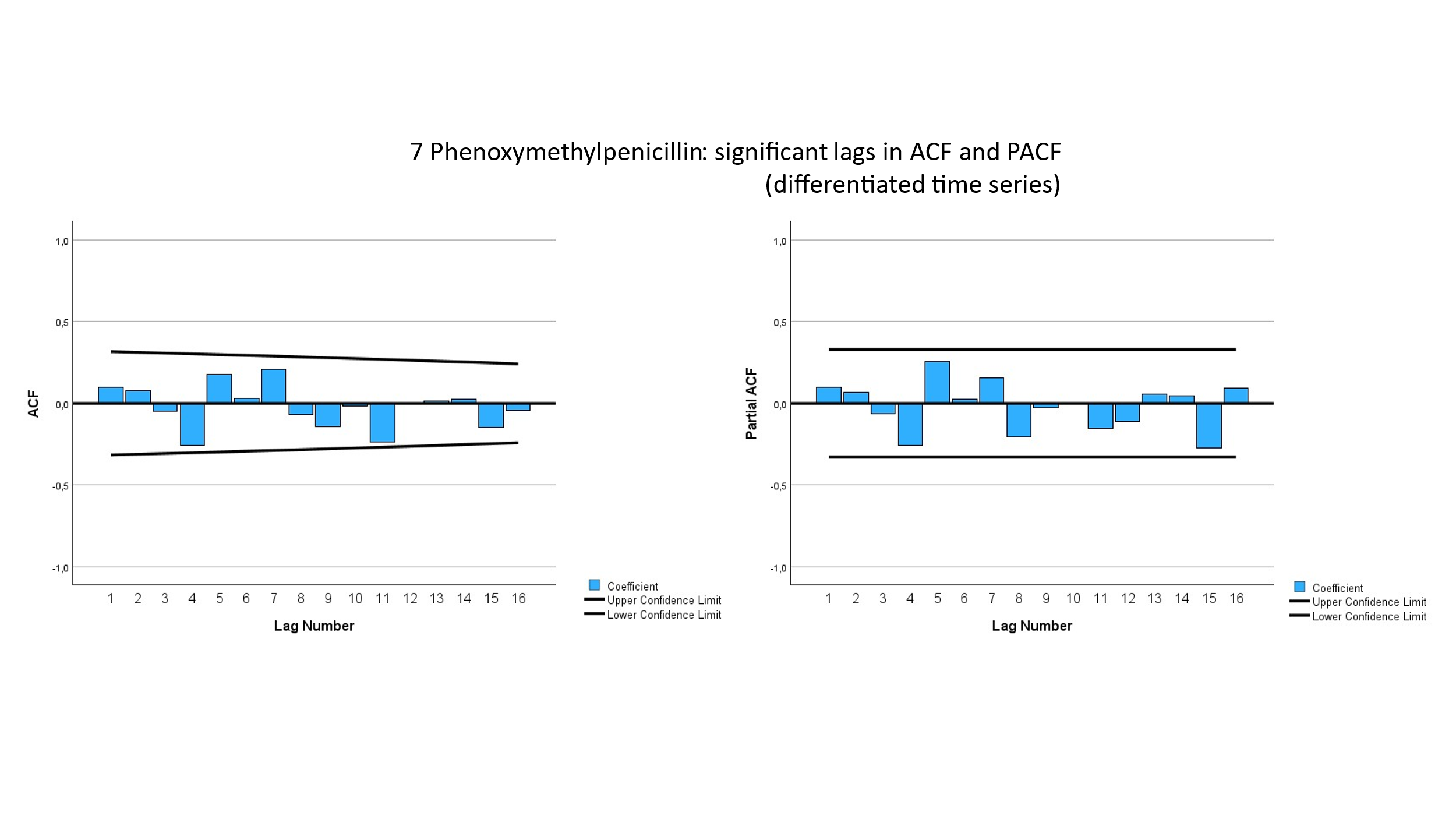


***Fig. S15****: Autocorrelation parameters ACF and PACF of the original time series, before performing any differentiation, for sulfamethoxazole-trimethoprim.*


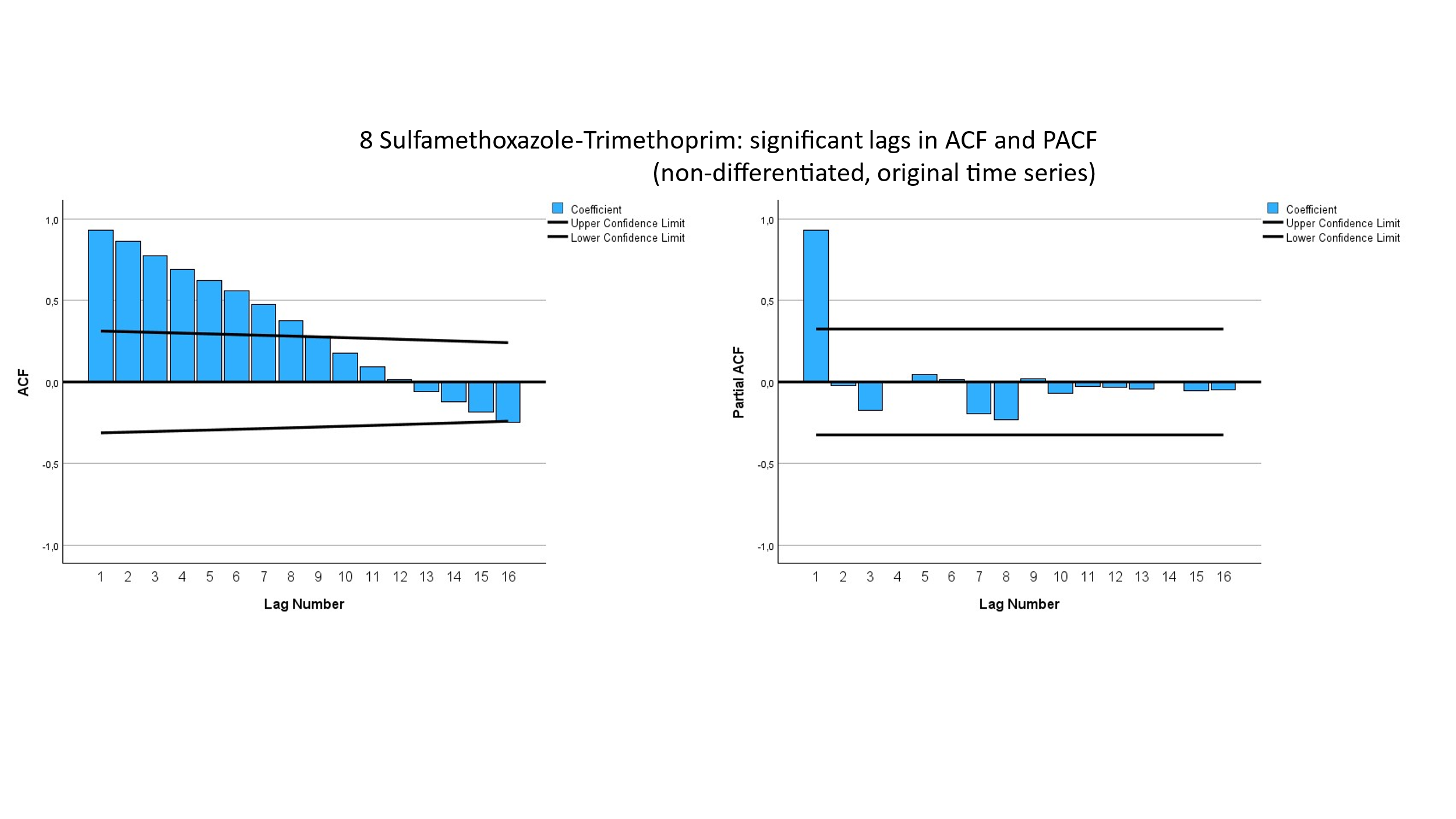


***Fig. S16****: Autocorrelation parameters ACF and PACF after performing one differentiation for sulfamethoxazole-trimethoprim. Significant lags, characterised by exceeding the black line, are considered as possible values for the respective ARIMA-model.*

**
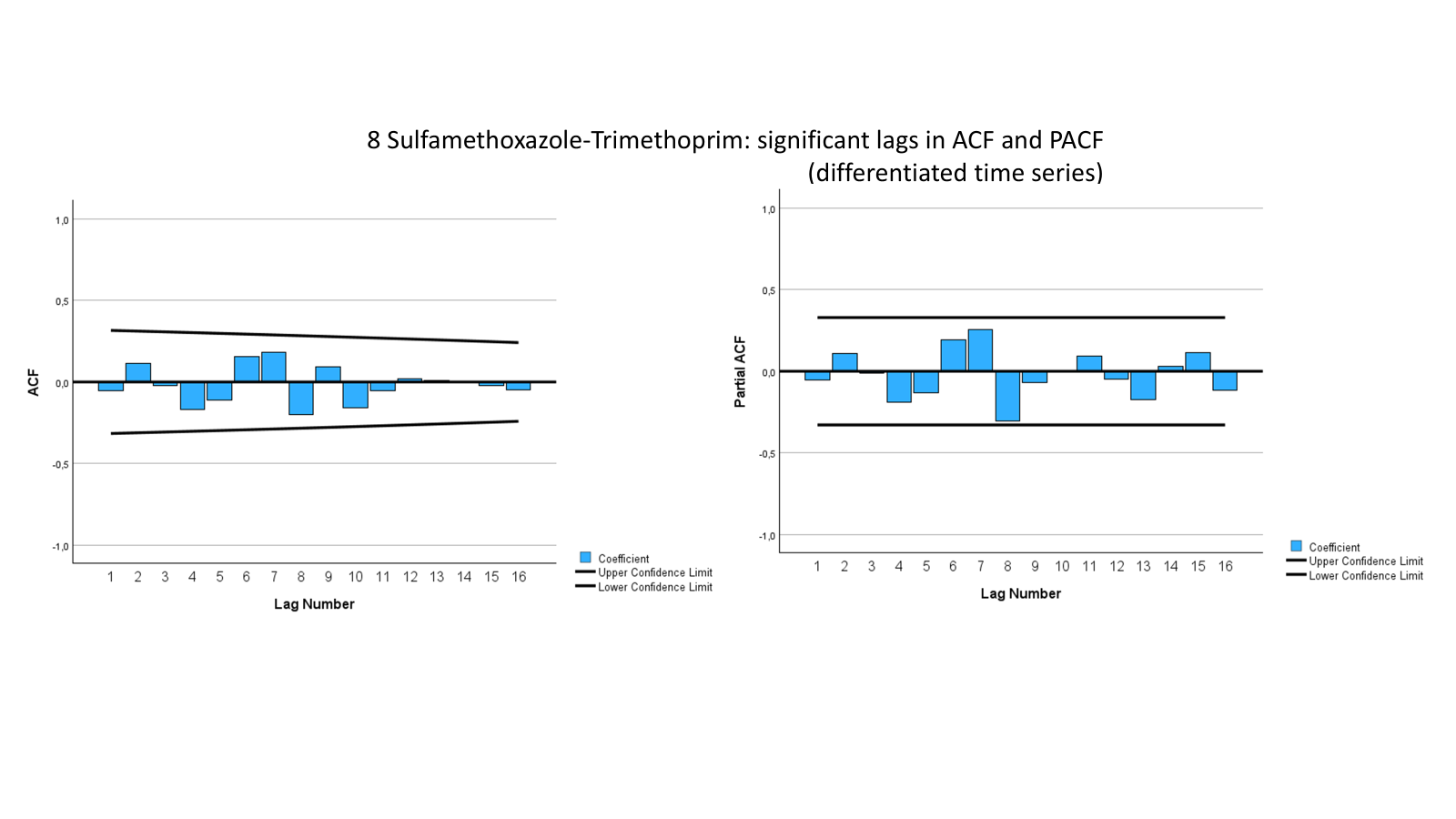
**

***Fig. S17****: Autocorrelation parameters ACF and PACF of the original time series, before performing any differentiation, for nitrofurantoin.*


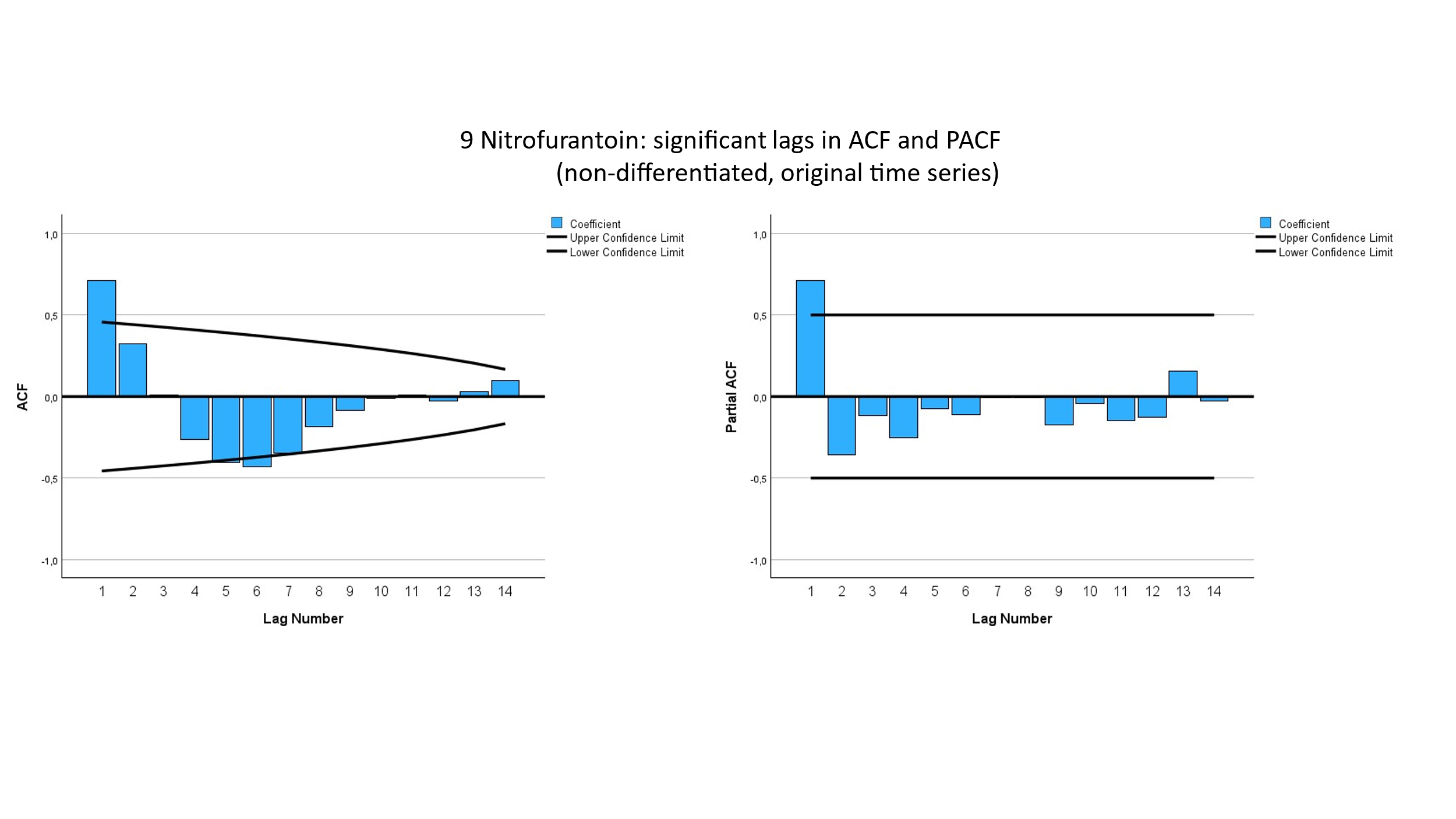


***Fig. S18****: Autocorrelation parameters ACF and PACF after performing one differentiation for nitrofurantoin. Significant lags, characterised by exceeding the black line, are considered as possible values for the respective ARIMA-model.*


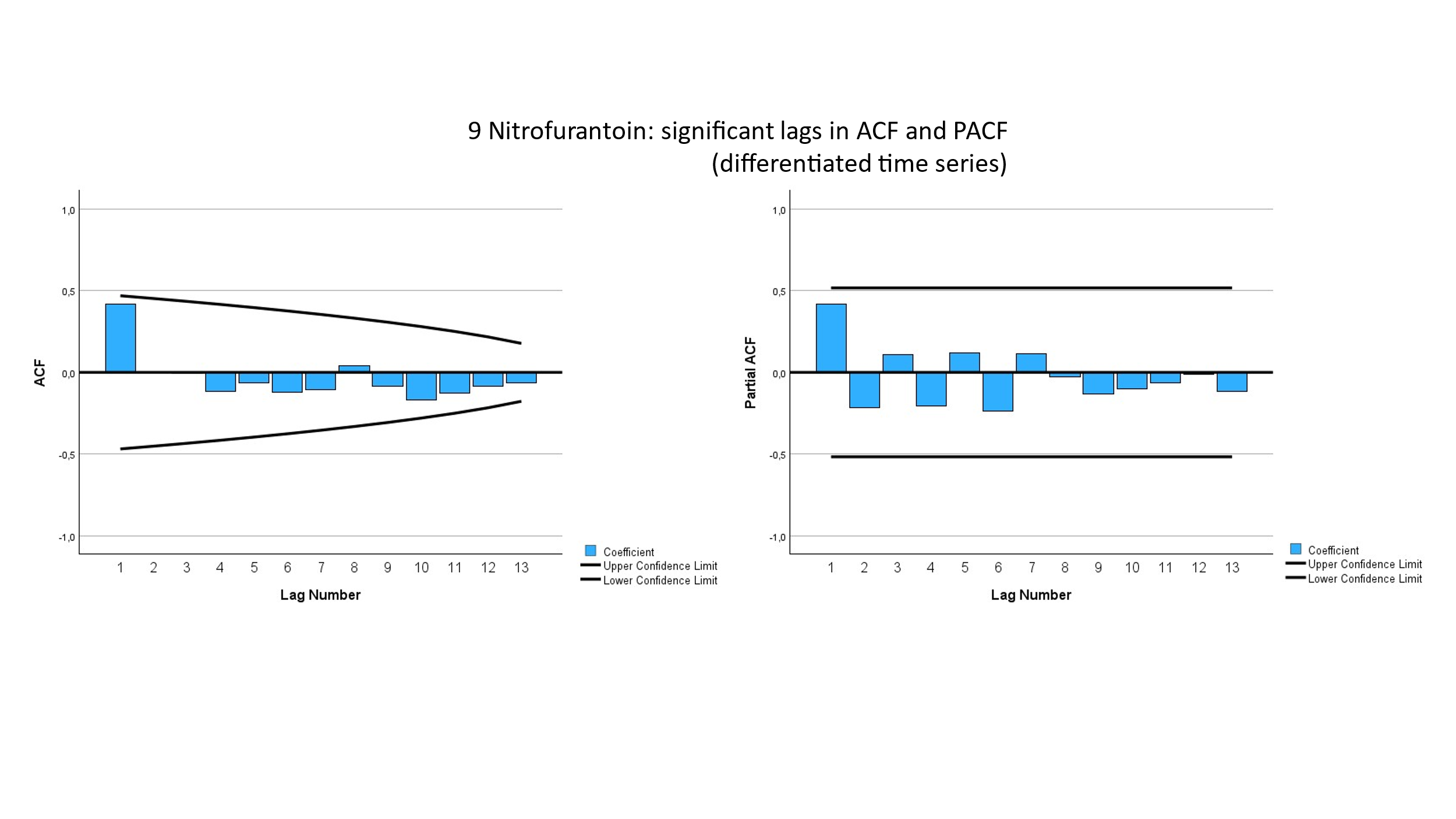


***Fig. S19****: Autocorrelation parameters ACF and PACF of the original time series, before performing any differentiation, for ciprofloxacin.*


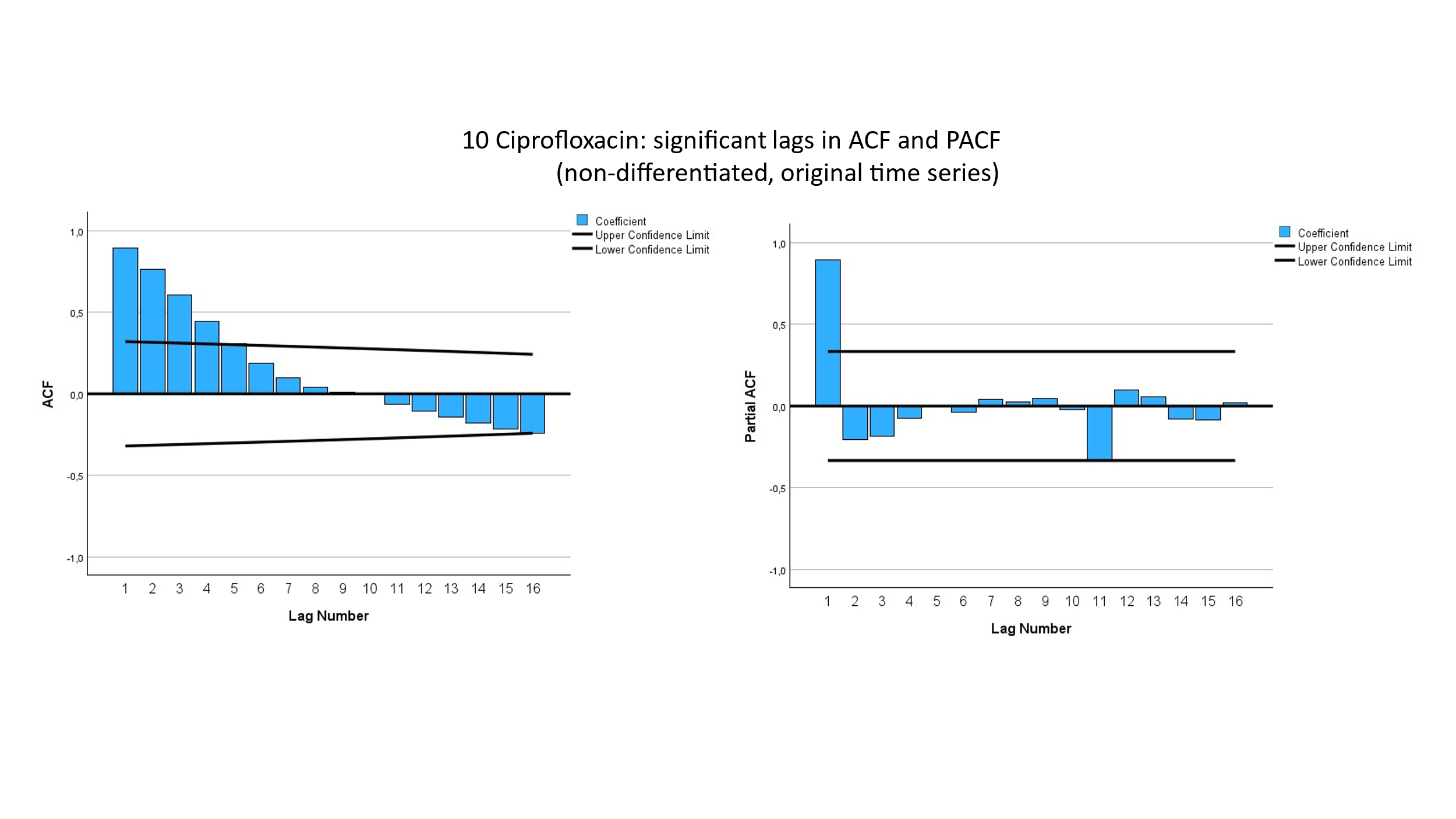


***Fig. S20****: Autocorrelation parameters ACF and PACF after performing one differentiation for ciprofloxacin. Significant lags, characterised by exceeding the black line, are considered as possible values for the respective ARIMA-model.*

**
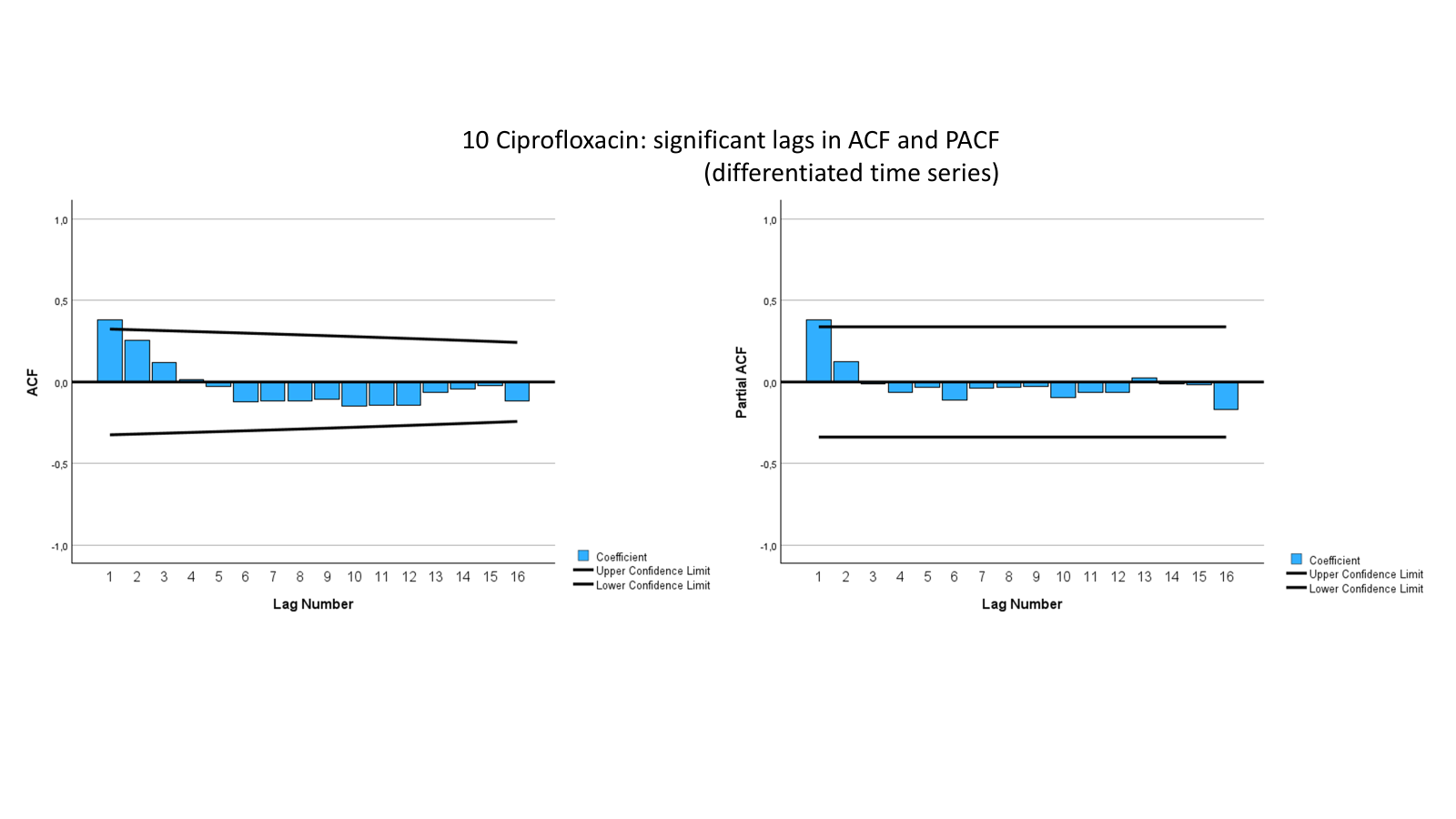
**

***Fig. S21:*** *Predictions of future DDD-prescriptions of fitting ARIMA-models for cefuroxime axetil. Models considered as suitable are ARIMA(0,1,0) in a), ARIMA(1,1,1) in b), ARIMA(1,1,8) in c) and ARIMA(1,1,11) in d). The model which is considered as best-fitting is in a green box, being most reliable for forecasting future demand.*


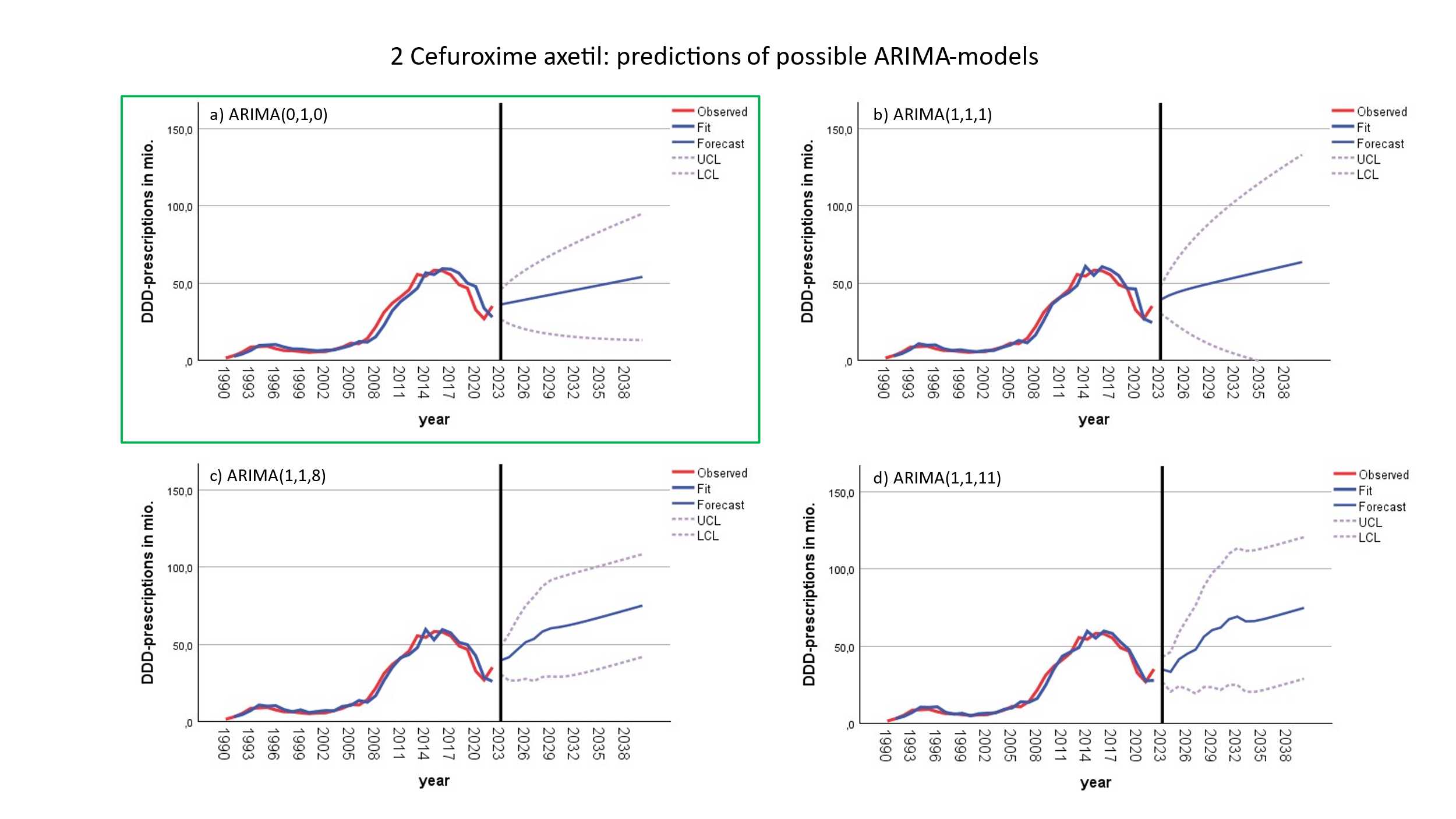


***Fig.22:*** *Predictions of future DDD-prescriptions of fitting ARIMA-models for doxycycline. Models considered as suitable are ARIMA(0,1,0) in a) and ARIMA(8,1,0) in b). The model which is considered as best-fitting is in a green box.*


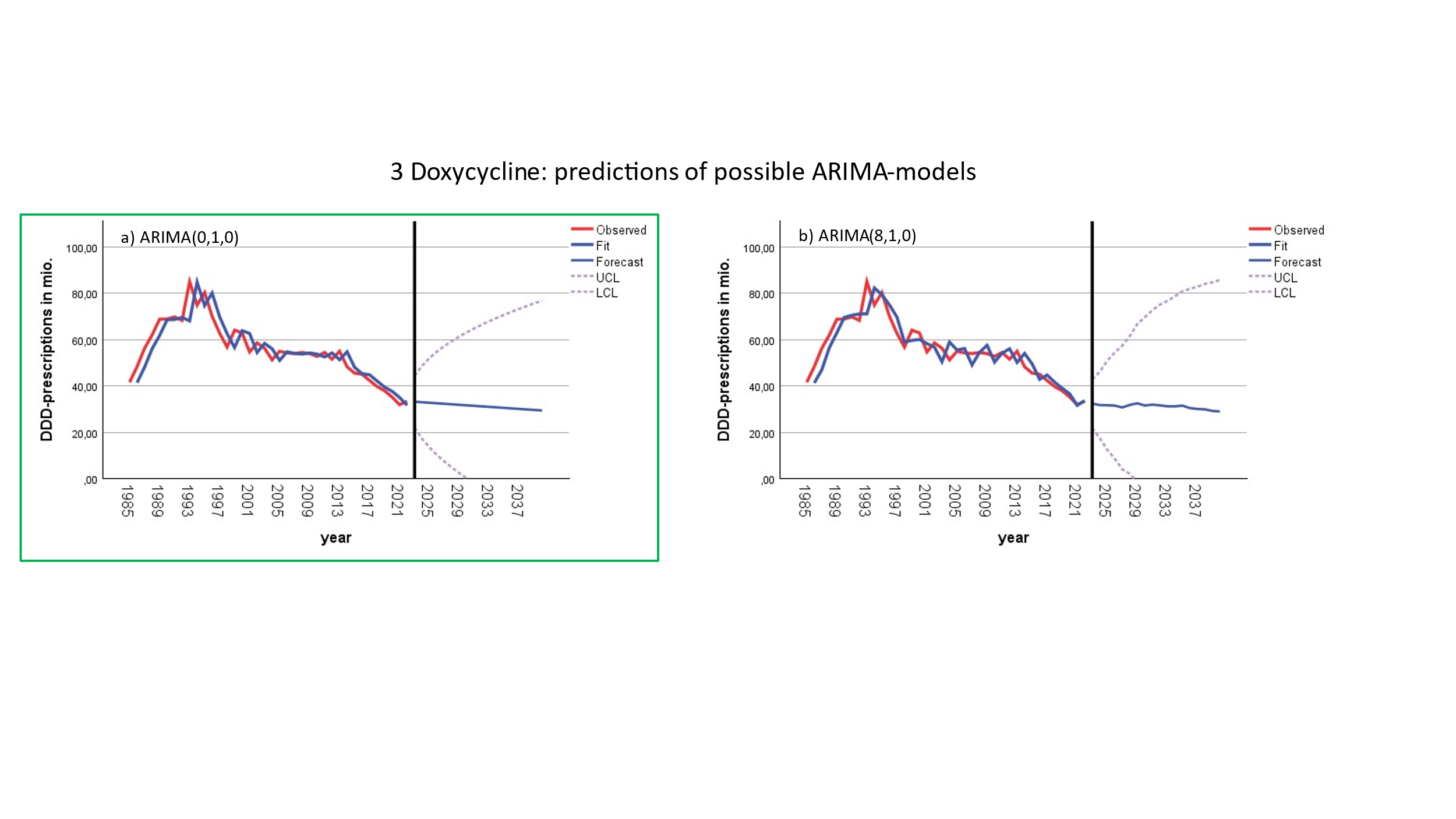


***Fig. S23:*** *Predictions of future DDD-prescriptions of fitting ARIMA-models for clindamycin. The model which is considered as best-fitting is shown.*


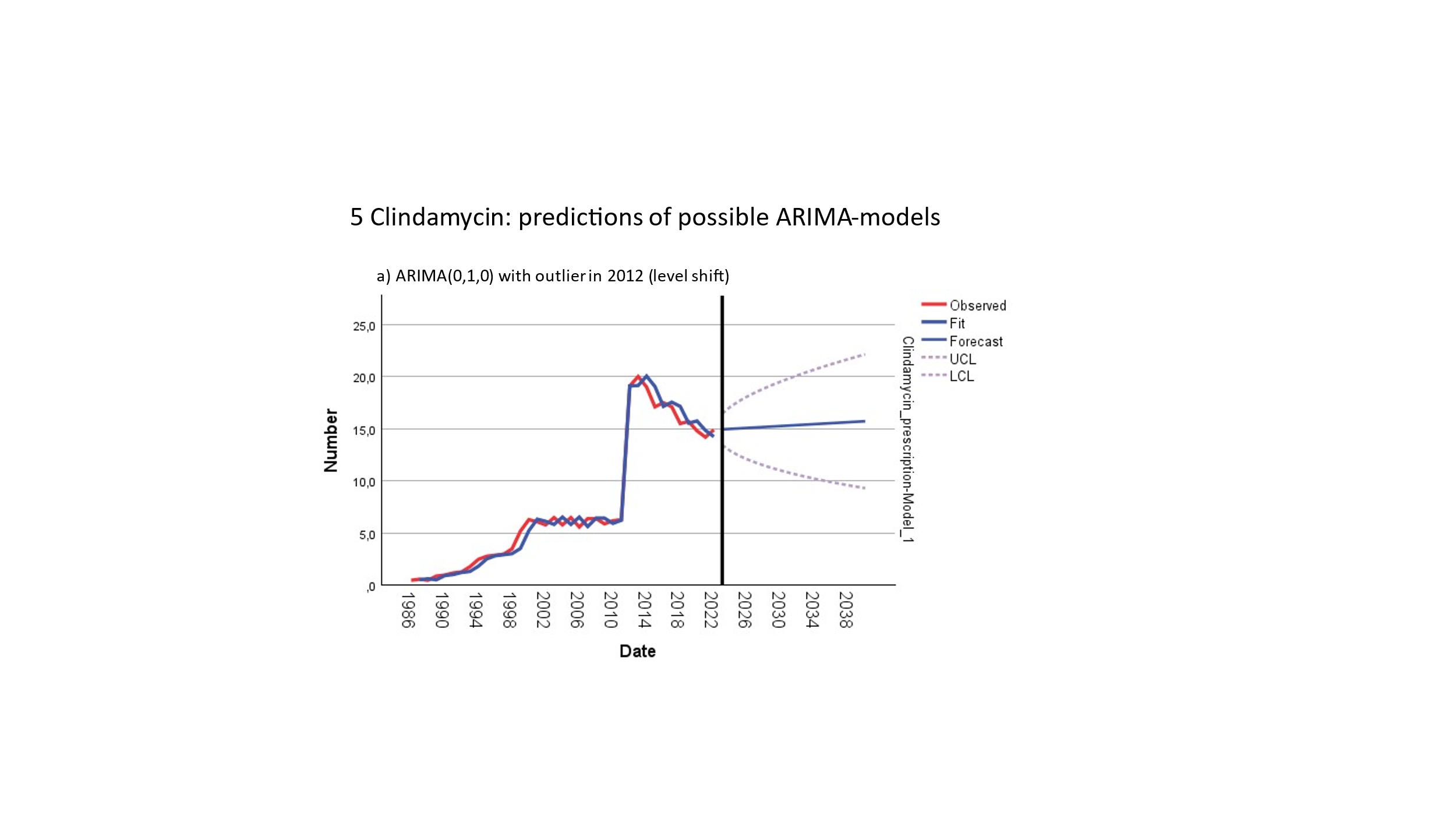


***Fig. S24:*** *Predictions of future DDD-prescriptions of fitting ARIMA-models for azithromycin. The model which is considered as best-fitting is shown.*


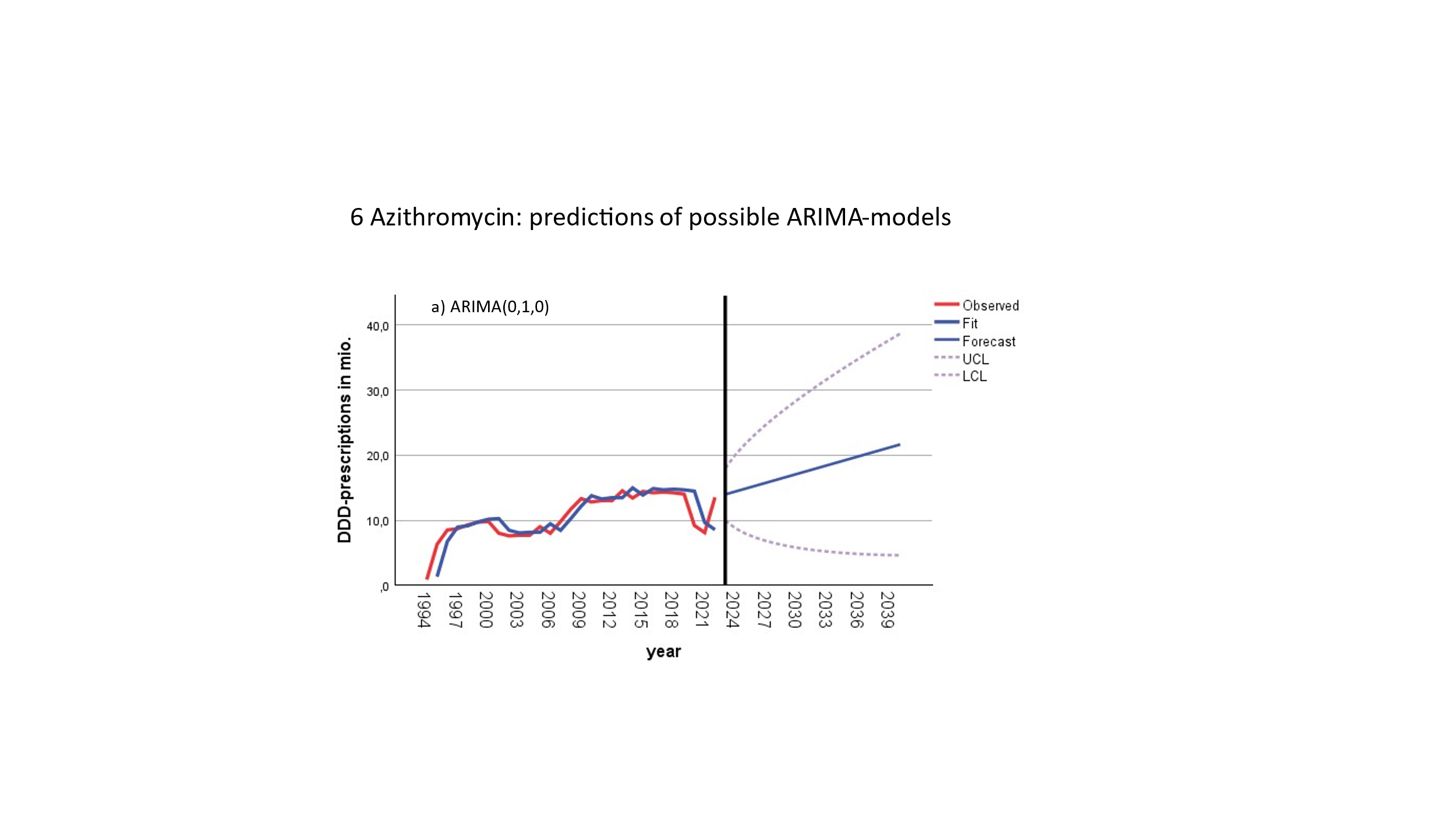


***Fig. S25:*** *Predictions of future DDD-prescriptions of fitting ARIMA-models for phenoxymethylpenicillin. The model which is considered as best-fitting is shown.*


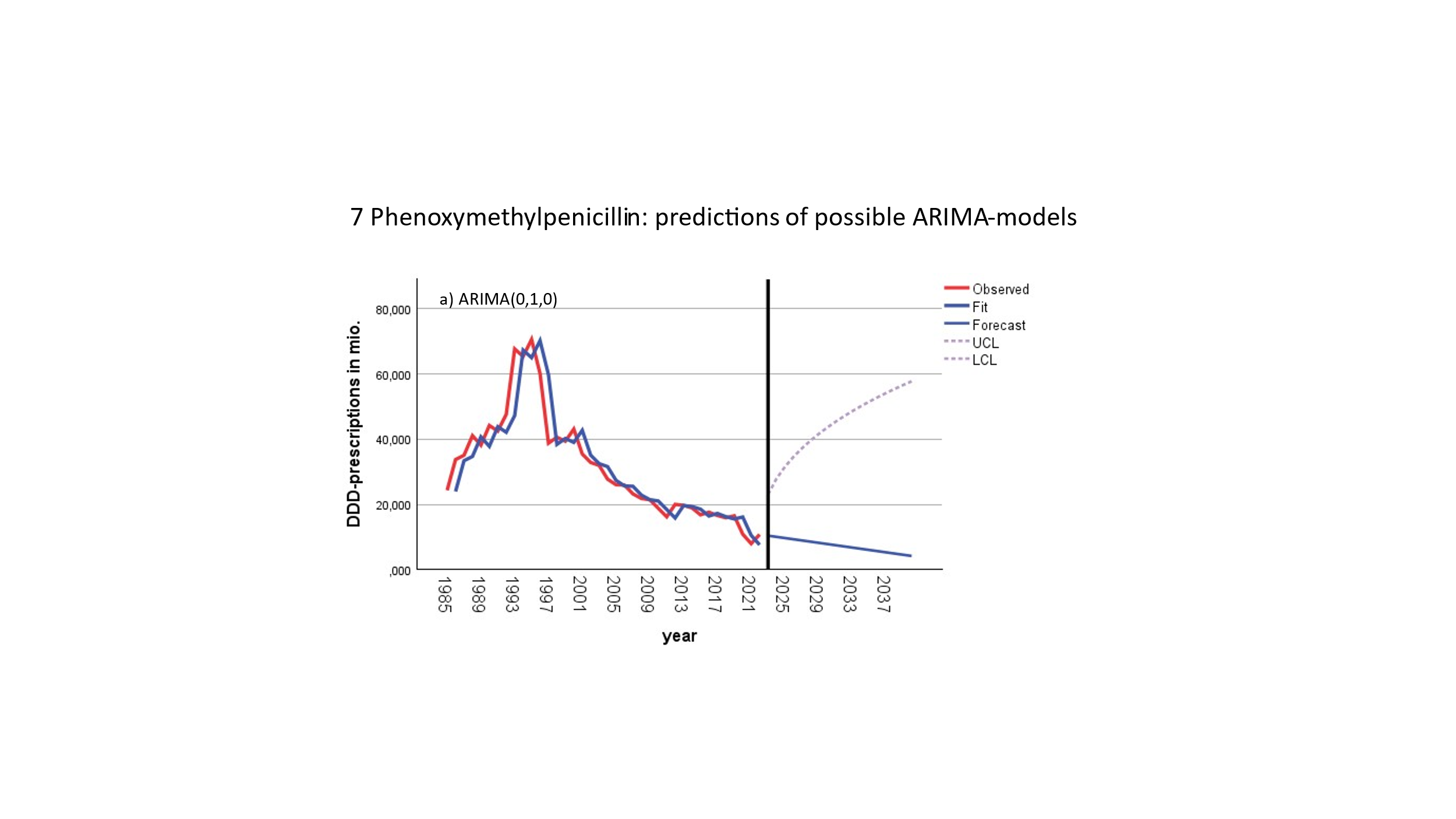


***Fig. S26:*** *Predictions of future DDD-prescriptions of fitting ARIMA-models for sulfamethoxazole-trimethoprim. The model which is considered as best-fitting is shown.*


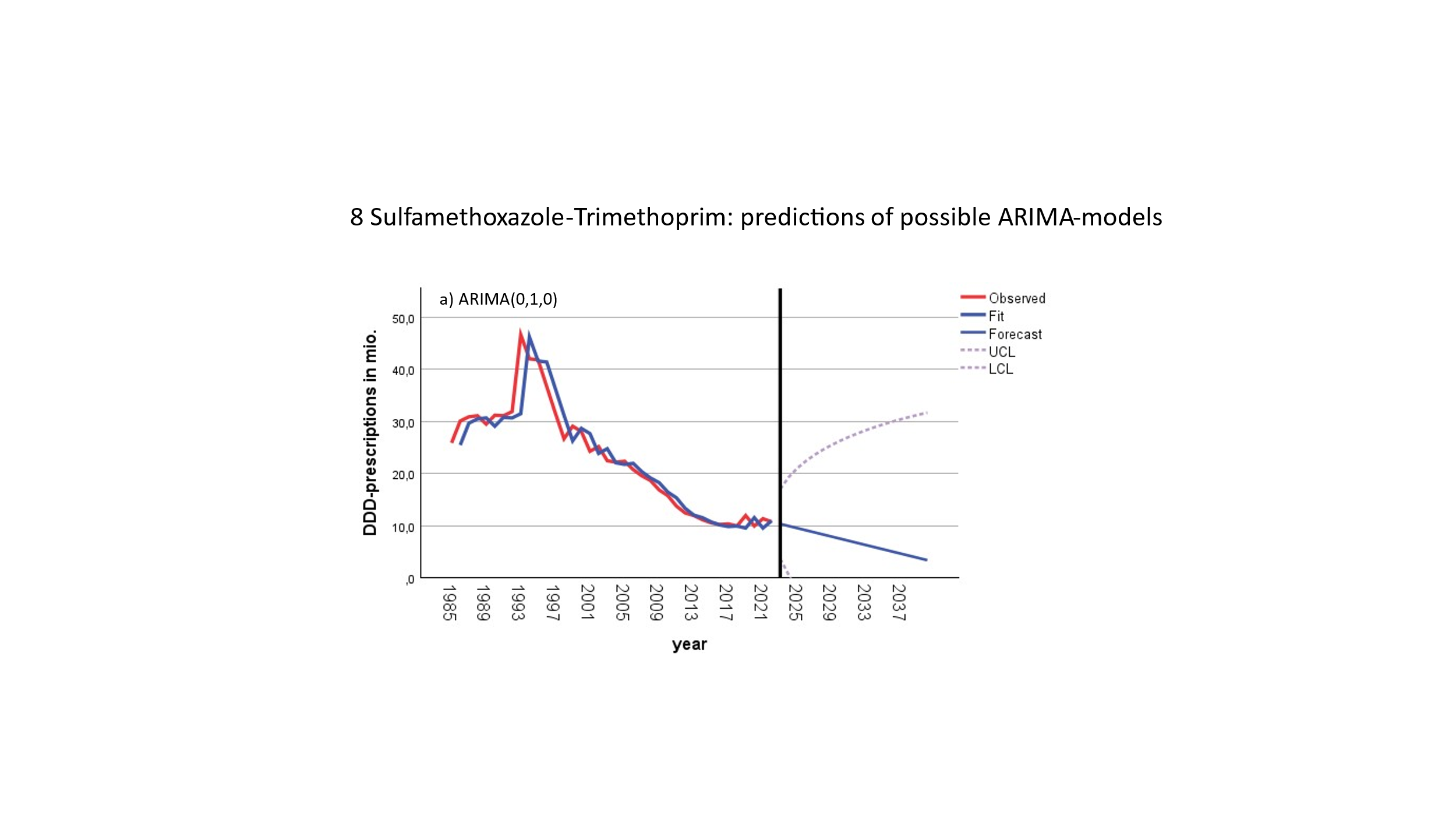


***Fig. S27:*** *Predictions of future DDD-prescriptions of fitting ARIMA-models for nitrofurantoin. The model which is considered as best-fitting is shown.*


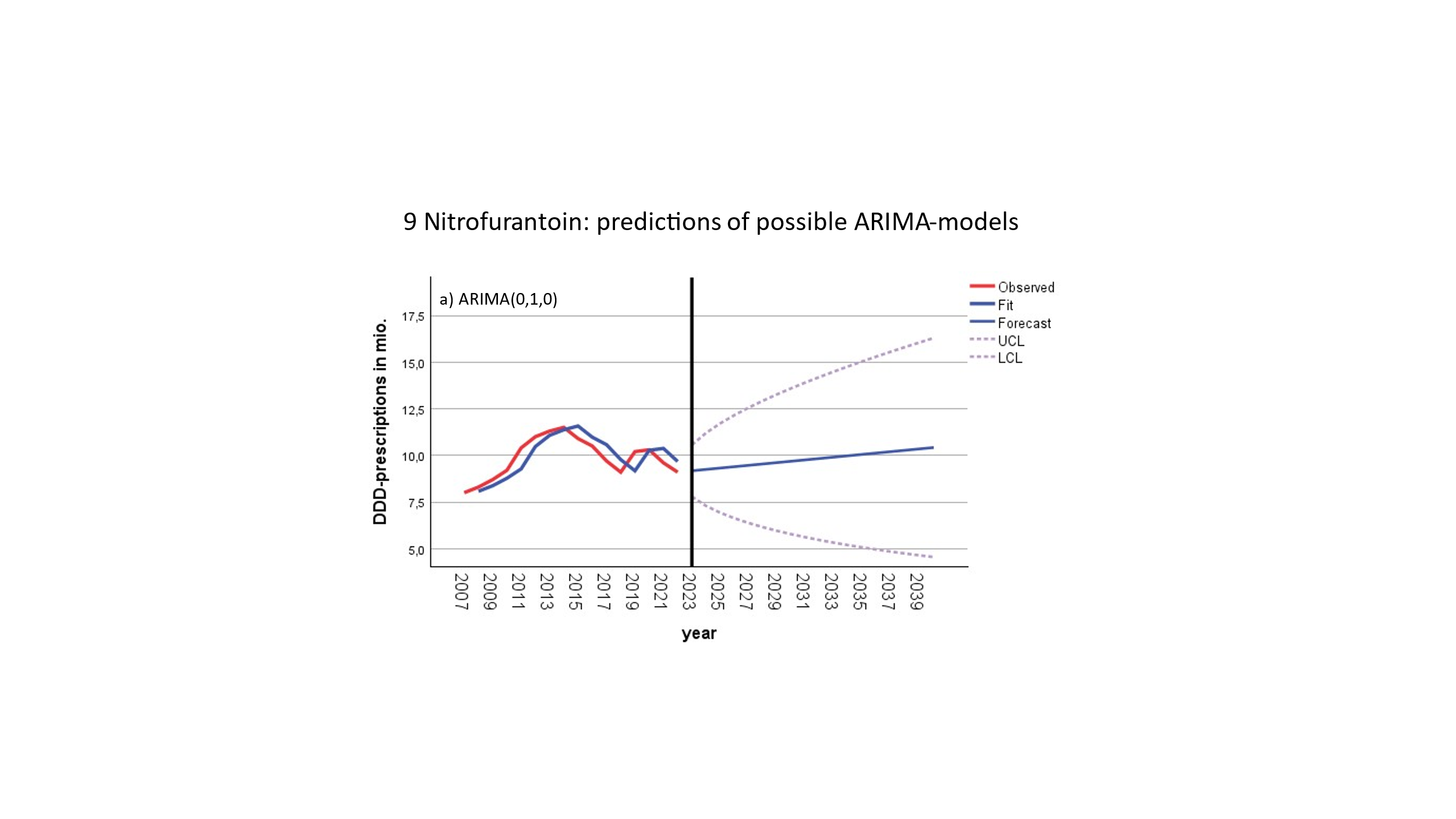


***Fig. S28:*** *Predictions of future DDD-prescriptions of fitting ARIMA-models for ciprofloxacin. The model which is considered as best-fitting is shown.*


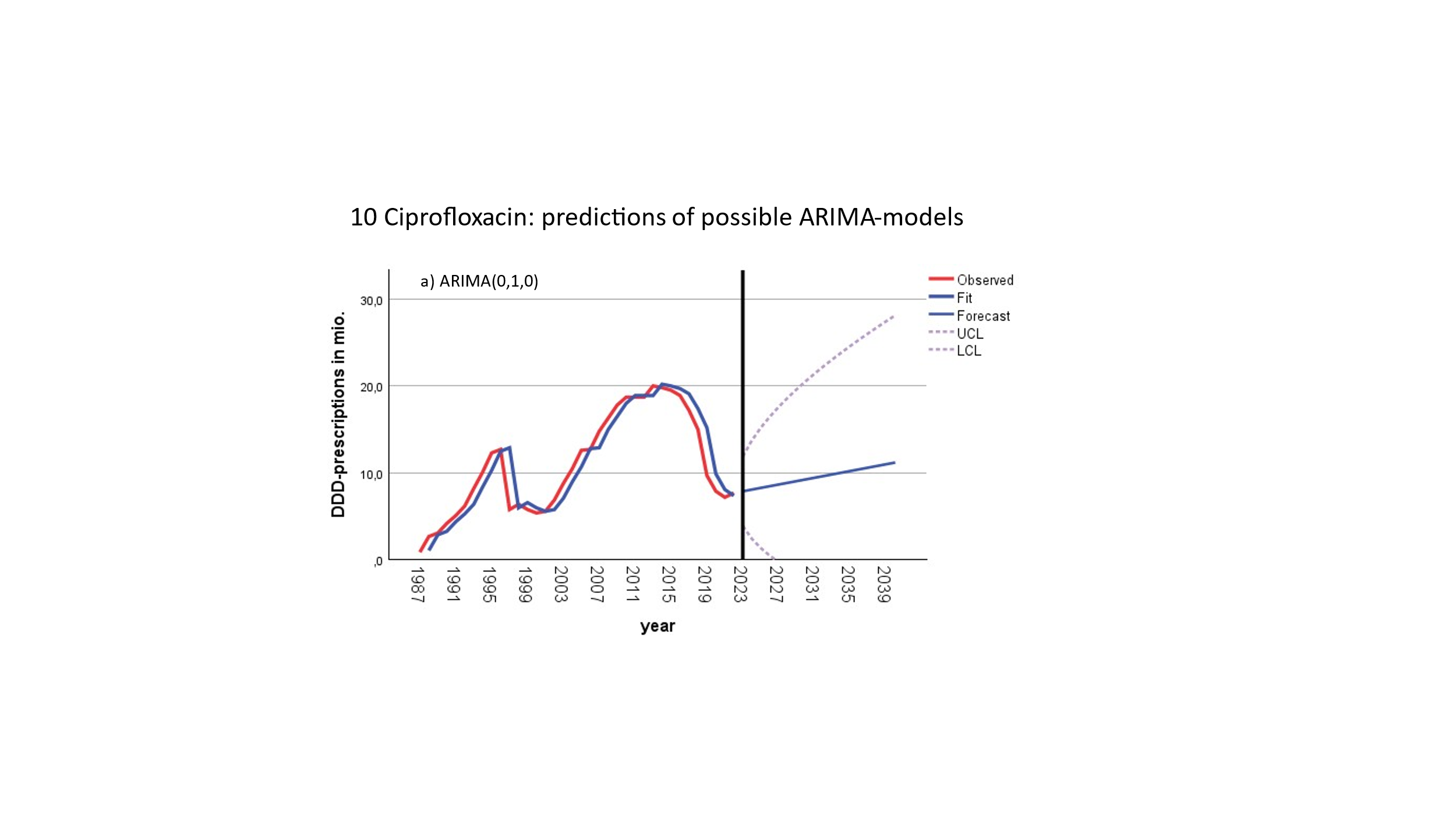

Supplement: Supplementary file 1 — Supplementary file1 (DOCX 8.30 MB) [file 210_2024_3721_MOESM1_ESM.docx]
